# The Global Response of *Cronobacter sakazakii* Cells to Amino Acid Deficiency

**DOI:** 10.3389/fmicb.2018.01875

**Published:** 2018-08-14

**Authors:** Si Chen, Qing Zhou, Xin Tan, Ye Li, Ge Ren, Xiaoyuan Wang

**Affiliations:** ^1^State Key Laboratory of Food Science and Technology, Jiangnan University, Wuxi, China; ^2^Key Laboratory of Industrial Biotechnology, Ministry of Education, School of Biotechnology, Jiangnan University, Wuxi, China; ^3^International Joint Laboratory on Food Safety, Jiangnan University, Wuxi, China

**Keywords:** *Cronobacter sakazakii*, colanic acids, exopolysaccharide, amino acid deficiency, transcriptomic analysis

## Abstract

*Cronobacter* species can cause necrotizing enterocolitis and meningitis in neonates and infants, their infection is closely relevant to their responses to extreme growth conditions. In this study, the response of *Cronobacter* species to amino acid deficiency has been investigated. Four *Cronobacter* species formed smooth colonies when grown on the solid LB medium, but formed mucoid colonies when grown on the amino acid deficient M9 medium. When the mucoid colonies were stained with tannin mordant, exopolysaccharide around the cells could be discerned. The exopolysaccharide was isolated, analyzed, and identified as colanic acid. When genes *wcaD* and *wcaE* relevant to colanic acid biosynthesis were deleted in *Cronobacter sakazakii* BAA-894, no exopolysaccharide could be produced, confirming the exopolysaccharide formed in *C. sakazakii* grown in M9 is colanic acid. On the other hand, when genes *rcsA*, *rcsB*, *rcsC*, *rcsD*, or *rcsF* relevant to Rcs phosphorelay system was deleted in *C. sakazakii* BAA-894, colanic acid could not be produced, suggesting that the production of colanic acid in *C. sakazakii* is regulated by Rcs phosphorelay system. Furthermore, *C. sakazakii* BAA-894 grown in M9 supplemented with amino acids could not produce exopolysaccharide. Transcriptomes of *C. sakazakii* BAA-894 grown in M9 or LB were analyzed. A total of 3956 genes were differentially expressed in M9, of which 2339 were up-regulated and 1617 were down-regulated. When *C. sakazakii* BAA-894 was grown in M9, the genes relevant to the biosynthesis of exopolysaccharide were significantly up-regulated; on the other hand, the genes relevant to the flagellum formation and chemotaxis were significantly down-regulated; in addition, most genes relevant to various amino acid biosynthesis were also significantly regulated. The results demonstrate that amino acid deficiency has a global impact on *C. sakazakii* cells.

## Introduction

*Cronobacter* species are food-borne opportunistic pathogens that can cause necrotizing enterocolitis and meningitis in neonates and infants (Feeney et al., 2014; Forsythe, 2017). *Cronobacter sakazakii*, *C. dublinensis*, *C. malonaticus*, and *C. turicensis* are the common *Cronobacter* isolates, especially *C. sakazakii* which has been extensively studied (Zhang et al., 2014; Liang et al., 2017; Li et al., 2017). *C. sakazakii* is much more resistant than other Enterobacteriaceae to environmental stresses (Dancer et al., 2009), which might be associated with its pathogenesis (Alvarez-Ordóñez et al., 2014). Biofilm formation (Jung et al., 2013), exopolysaccharide production, and lipopolysaccharide structure modification (Wang and Quinn, 2010; Zhang et al., 2010; Wiederschain, 2011; Cai et al., 2013; Wang et al., 2015; Liu et al., 2016) are various strategies for *C. sakazakii* to cope with the environmental stress.

Bacteria tend to use abundant nutrients in the surroundings for its maximum growth but could also survive in the environment lacking enough nutrients. The shortage of amino acids, the most important nitrogen source for bacteria, can lead to a coordinated regulation of metabolism, which is very important for the bacteria adapting to the new environment (Kuroda et al., 1999). In *Escherichia coli*, amino acid starvation results in the phosphorylation of the response regulator NtrC, which activates RelA (Shyp et al., 2012). RelA is responsible for synthesizing the ppGpp, which causes the stringent response (Brown et al., 2014). Amino acid starvation can result in the coordinate inhibition of a variety of metabolic activities in *E. coli*, such as fatty acid and phospholipid biosynthesis (Podkovyrov and Larson, 1996). Amino acid starvation in *C. sakazakii* has not been investigated yet. Understanding the responses of *C. sakazakii* to amino acid starvation might provide important information for *C. sakazakii* infection.

In this study, the response of *C. sakazakii* BAA-894 cells to amino acid deficiency has been investigated. When amino acids were not available, the significantly regulated genes in *C. sakazakii* cells include not only the ones relevant to various amino acid biosynthesis, but also the ones relevant to the biosynthesis of exopolysaccharide, flagellum, and chemotaxis; the results suggest that amino acid deficiency has a global impact on *C. sakazakii* cells.

## Materials and Methods

### Bacterial Strains and Growth Conditions

*Cronobacter* species were grown at 30°C in Luria-Bertani (LB) and M9 media. Luria-Bertani medium contains 5 g/l yeast extract, 10 g/l tryptone, and 10 g/l NaCl. The M9 medium contains 17.1 g/l Na_2_HPO_4_⋅12H_2_O, 3 g/l KH_2_PO_4_, 4 g/l glucose, 1 g/l NH_4_Cl, 0.5 g/l NaCl, 0.24 g/l MgSO_4_, and 0.011 g/l CaCl_2_. Fifteen important amino acids, L-aspartic acid (Asp), L-glutamic acid (Glu), L-serine (Ser), L-histidine (His), L-glycine (Gly), L-threonine (Thr), L-arginine (Arg), L-alanine (Ala), L-cysteine (Cys), L-valine (Val), L-methionine (Met), L-phenylalanine (Phe), L-isoleucine (Ile), L-leucine (Leu), and L-lysine (Lys) with the final concentration of 0.5 or 5 mM were added in M9 medium when necessary.

### Identification of Exopolysaccharide

Exopolysaccharide was identified by the tannin mordant staining method (Ren et al., 2016). *Cronobacter* species were grown on agar media of LB, M9, or M9 with amino acids at 30°C for 2 days. The bacterial colonies were fixed on slides by smearing and drying, and then treated with the fuchsin solution for 3 min. The fuchsin solution was composed of 0.3 g basic fuchsin, 10 ml 95% ethanol, and 90 ml 5% phenol. Thr freshly prepared mordant solution was then added, incubated for 3 min, and washed with distilled water. Mordant solution contains 2 volumes of 0.3 g/l FeCl_3_, 2 volumes of 1.5 g/l tannins, and 5 volumes of 2 g/l saturated potassium aluminum sulfate solution. Prior to examination of the samples under the oil immersion lens, 1% methylene blue was used to counterstain for 1 min. The cells were stained red and the exopolysaccharide blue.

### Construction of *Cronobacter sakazakii* Mutants

The lambda Red recombinase system was used to delete genes in *C. sakazakii* BAA-894 (Datsenko and Wanner, 2000; Serra-Moreno et al., 2006). To delete the genes *ESA_RS05320* and *ESA_RS05325* (homologs of *E. coli wcaD* and *wcaE*, respectively), their upstream and downstream fragments were PCR amplified using the primer pairs *wcaDE*-U-F/*wcaDE*-U-R and *wcaDE*-D-F/*wcaDE*-D-R, respectively. The PCR product of the upstream fragment was digested with *Sac*I and *EcoR*I, while that of the downstream fragment was digested with *BamH*I and *Nde*I. The DNA fragment *loxP-kan-loxP* containing the kanamycin resistance gene *kan* was amplified from pDTW202 using the primer pairs of *kan-loxP-F*/*kan-loxP-R*, and digested with *EcoR*I and *BamH*I. The three digested fragments were inserted into pBlueScript II SK (+) which was digested with *Sac*I and *Nde*I, resulting the plasmid pBS-kan-*wcaDE* which carries the knockout DNA fragment *wcaDE*U-*loxP-kan-loxP-wcaDE*D. The knockout DNA fragment was then PCR amplified using the primer pairs *wcaDE*-U-F/*wcaDE*-D-R, and transferred into BAA894 cells containing the plasmid pKD46 by electroporation, resulting in the replacement of *ESA_RS05320* and *ESA_RS05325* with *loxP-kan-loxP* in the chromosome. After the correct transformants were selected by growing cells on LB plates containing kanamycin, the temperature sensitive plasmid pKD46 was cured by growing the cells at 42°C. The plasmid pKD-Cre was then introduced into the cells, and the *kan* gene in the chromosome was removed by the *loxP* recombinase Cre. The temperature sensitive plasmid pKD-Cre was cured by growing at 42°C, resulting in the mutant strain Δ*wcaDE*. The successful insertion and deletion of *kan* in the chromosome were confirmed by PCR analysis. Similarly, the genes *ESA_RS05720*, *ESA_RS04445*, *ESA_RS04440*, *ESA_RS04450*, or *ESA_RS14450* (homologs of *E. coli rcsA*, *rcsB*, *rcsC*, *rcsD*, or *rcsF*, respectively) in the chromosome of *C. sakazakii* BAA894 were removed, resulting in the mutants Δ*rcsA*, Δ*rcsB*, Δ*rcsC*, Δ*rcsD*, or Δ*rcsF*, respectively. All of the strains and plasmids used in this study are listed in **Table 1**. All primers used in this study are listed in **Table 2**. Since there are no selection markers left on the chromosomes of these mutant strains, they could grow in medium without the addition of any antibiotics.

**Table 1:** Bacterial strains and plasmids used in this study.

Strains and plasmids	Description	Source
MG1655	Wild-type *E. coli*	ATCC
BAA-894	Wild-type *C. sakazakii*	ATCC
ATCC 51329	Wild-type *C. muytjensii*	ATCC
DSM18703	Wild-type *C. turicensis*	DSM
DSM18705	Wild-type *C. dublinensis*	DSM
Δ*wcaDE*	*wcaD* and *wcaE* deletion mutant of BAA-894	This study
Δ*rcsA*	*rcsA* deletion mutant of BAA-894	This study
Δ*rcsB*	*rcsB* deletion mutant of BAA-894	This study
Δ*rcsC*	*rcsC* deletion mutant of BAA-894	This study
Δ*rcsD*	*rcsD* deletion mutant of BAA-894	This study
Δ*rcsF*	*rcsF* deletion mutant of BAA-894	This study
pWSK29	Low copy vector	Cai et al., 2013
pKD46	ParaBγβ exo, Repts, AmpR	Datsenko and Wanner, 2000
pKD-Cre	ParaB cre, Repts, AmpR	Han et al., 2013
pBlueScript II SK+	Cloning vector, ColE1, *lacZ*, AmpR	Stratagene
pDTW202	loxPLE-*kan*-loxPRE, AmpR, KanR	Han et al., 2013


**Table 2:** Primers for PCR amplification used in this study.

Primers	Nucleotide sequences (5′→3′)	Restriction sites
*wcaDE*-U-F	ACTGAGCTCGCGCAGGAAGTGCTCAATAA	*Sac*I
*wcaDE*-U-R	ACTGAATTCCGAACCCTCTGTGCCTAAATC	*EcoR*I
*wcaDE*-D-F	ACTGGATCCGCCATCAGGCCATTTTCTTC	*BamH*I
*wcaDE*-D-R	ACTCATATGATCGGTCAGGTCGCCATAGT	*Nde*I
*rcsA*-U-F	CATGAGCTCGGCTAACCAGGAATAATCTCA	*Sac*I
*rcsA*-U-R	CAGGGATCCGCGGCGTAACAATAAGTAAA	*BamH*I
*rcsA*-D-F	CACGAATTCCAGGCTTCTTCCAGAGTTT	*EcoR*I
*rcsA*-D-R	CACTCTAGATTCACCAGCGACCAGTAT	*Xba*I
*rcsB*-U-F	CAGCTCGAGCCTGATTACCGACGATGAAAA	*Xho*I
*rcsB*-U-R	GCCCATATGCATCAGTAGCCAGAAGAAATCG	*Nde*I
*rcsB*-D-F	GCAAAGCTTACTCACCGACAACATTCACCC	*Hind*III
*rcsB*-D-R	ACTTCTAGATCCGTCACCACCACGCAGATT	*Xba*I
*rcsC*-U-F	CAGAGTACTAAGCGAACGAGCGTATCAC	*Sca*I
*rcsC*-U-R	CCGTCTAGAAAAGGAATGCCGTTAAGGTAG	*Xba*I
*rcsC*-D-F	CACATATGGTGTATGCCGACAGGGTT	*Nde*I
*rcsC*-D-R	CACTCGAGATGCCACAGCCATAGAAAA	*Xho*I
*rcsD*-U-F	CATTCTAGACGACAGGGTTCGCAAGAC	*Xba*I
*rcsD*-U-R	CATGAGCTCGATGCCACAGCCATAGAA	*Sac*I
*rcsD*-D-F	AAAGTCGACCCATCGTCACCAGCAACA	*Sal*I
*rcsD*-D-R	GCAGGATCCAGCCGAATAATCCAACAG	*BamH*I
*rcsF*-U-F	ACTAGTACTAGTGGGCGTAATTGTGGG	*Sca*I
*rcsF*-U-R	CAGTCTAGAGTGGCTTATTGAATCAGGAG	*Xba*I
*rcsF*-D-F	CCAGAATTCAGCGTTCCGACCAAATGA	*EcoR*I
*rcsF*-D-R	CACAAGCTTCAGATATTGGCTAGTACGCATG	*Hind*III
*Kan*-loxP-F	CACTCTAGAAATACGACTCACTATAGGGCG	*Xba*I
*Kan*-loxP-R	ACCCATATGGCGCAATTAACCCTCACTAAAG	*Nde*I


*The restriction enzyme sites were underlined.*

### Purification of Exopolysaccharide

Exopolysaccharide was purified from *C. sakazakii* BAA-894 according to the published procedure (Navasa et al., 2009) with minor modification. Cells were grown in M9 medium at 30°C and 200 rpm for 24 h and harvested by centrifugation. The supernatant was collected and mixed with 2 volumes of ice-cold anhydrous ethanol. After precipitating at 4°C for 3 h, the solid product was resuspended in water to a concentration of 100 mg/l. Nucleic acids were precipitated by adding 0.75% streptomycin sulfate (w/v), and removed by centrifugation at 13,800 × *g* for 30 min. Lipopolysaccharide was precipitated by treating with 1% glacial acetic acid (v/v) at 100°C for 2 h, and removed by centrifugation at 13,800 × *g* for 15 min at 4°C (Ren et al., 2016). The supernatant was extracted with 1 volume of chloroform–methanol (2:1, v:v), and the aqueous phase was collected, dialyzed against distilled water (MWCO, 3600) for 48 h, and then lyophilized.

### Colanic Acid Analysis by High-Performance Liquid Chromatography

Two milligram colanic acid (CA) sample was mixed with 1 ml 4 M trifluoroacetic acid solution in a tube and heated at 110 °C for 1 h; 1 ml methanol was then added in the mixture and dried under N_2_. This process was repeated twice. The sample was then resolved in 1 ml deionized water and analyzed by high-performance liquid chromatography (HPLC) with an ion-exchange column (HPLC ICS-5000; Dionex Corporation, United States). A CarboPac PA20 column and a pulsed amperometry detector were used. Mobile phases used were 6.5 mM NaOH in the first 21 min, 6.5 mM NaOH and 50–200 mM NaAc for 21–30 min, and 200 mM NaOH for 30-50 min. The flow rate was 0.5 ml/min.

### Quantitative Determination of Colanic Acid Production

*Cronobacter sakazakii* BAA-894 grown in liquid medium at 30°C for 48 h, and samples were collected at 12, 24, 36, and 48 h, then heated in a water bath at 90°C for 20 min to denature enzymes and release CA. After cooling, a 3 ml sample was centrifuged at 13,800 × *g* for 20 min, the supernatant was mixed with 2 volumes of ice-cold anhydrous ethanol, and incubated at 4°C for 3 h. The precipitate was collected by centrifugation at 10,000 × *g* for 15 min, dried and used for CA determination (Blumenkrantz and Asboe-Hansen, 1973). The sample was dissolved with 0.5 ml distilled water, treated with 2.5 ml sodium tetraborate/sulfuric acid solution (0.475 mg/l) and heated at 100°C for 3 min. After cooling, 100 μl hydroxy diphenyl (1.5 g/l) solution was added to the sample, and the absorbance at 526 nm was measured. The liquid medium was used as a control, and the standard curve was made using different concentrations of glucuronic acid. The number of cells used to determine the levels of CA production were normalized by serial dilution and the plating method (Ren et al., 2016).

### Quantification of Amino Acids

*Cronobacter sakazakii* BAA-894 cells were grown in medium of LB, M9, and M9 containing 0.5 or 5 mM amino acids at 30°C for 48 h, and centrifuged at 13800 × *g* for 20 min. The supernatant was mixed with the same volume of 10% Trichloroacetic acid to sediment at least 5 h. All samples were centrifuged at 13800 × *g* for 20 min again and filtered; the levels of 15 amino acids in the sample were then determined by HPLC (Agilent Technologies 1200 series, United States). The aqueous phase included 3.02 g/l sodium acetate, 200 μl/l trimethylamine and 5 ml/l tetrahydrofuran; the organic phase was composed of 3.02 g/l sodium acetate, 400 ml/l methanol and 400 ml/l acetonitrile. The pH of the two phases was adjusted to 7.2 with acetic acid.

### Whole Genome Transcriptional Analysis of *Cronobacter sakazakii*

For transcriptome analysis, *C. sakazakii* BAA-894 cells were cultivated in LB or M9 media at 30°C to the later exponential phase. BAA-894 cells were grown in LB for 6 h or in M9 for 9 h until they reached the mid-log phase. The samples were collected at 13,800 × *g* for 10 min and washed by using phosphate buffer solution. The whole libraries of RNA were prepared and sequenced using an Illumina HiSeq 2000 (BGI Shenzhen, China). The whole genome transcriptional analysis was established according to a published method (Marisch et al., 2013). The differentially expressed genes were determined based on their expression levels in the different strains (Mortazavi et al., 2008), and the KEGG (Kyoto Encyclopedia of Genes and Genomes) pathway and GO (Gene ontology) analysis were performed. The significance of the differences in gene expression was judged by the thresholds of false discovery rate (FDR ≤ 0.001) and the ratio of gene expression in the M9 medium versus the same gene in the LB medium using a logarithmic scale to base 2 (log_2_^R^ ≥ 1) (Kim and van de Wiel, 2008). The KEGG was used to perform the pathway enrichment analysis of the differentially expressed genes and to identify the signal transduction pathways or significantly enriched metabolic pathways based on the differentially expressed genes using the major public pathway-related database^1^ (Kanehisa et al., 2008). After correction with a significant level of 0.05, data analysis and the overrepresented biological process categories were generated. The GO analysis provides all GO terms that were significantly enriched in the list of differentially expressed genes. All differentially expressed genes were mapped to the GO terms in the database^2^, and the gene numbers for each term were calculated using GO-TermFinder^3^ (Ball et al., 2005); then, the hypergeometric test was used to find the significantly enriched GO terms in the input list of the differentially expressed genes. Transcriptome data have been deposited in the NCBI Sequence Read Archive database and linked with accession numbers SAMN08723122 (BAA-894 in M9 medium) and SAMN08723122 (BAA-894 in LB medium), respectively.

### RNA Extraction and Transcriptional Analysis Through RT-PCR

Total RNA was prepared using the Qiagen RNeasy Total RNA kit. For RT-PCR assays, 1 μg RNA sample was treated with RNase-free DNase I Set (Qiagen) and transcribed to cDNA with SuperScript II reverse transcriptase (Thermo Fisher) using random hexamers (Thermo Fisher) as primers. RT-PCR reactions were performed using the Applied Biosystems step one real-time PCR system (Life Technologies, California, United States). Relative transcript abundance was calculated using the ΔΔ*C*_T_ method (Livak and Schmittgen, 2001). Transcriptional data were normalized, using 16s RNA as a control. The transcription of a given gene was calculated as the difference in qPCR threshold cycles (Δ*C*_T_). As one PCR cycle represents a twofold difference in template abundance, fold change values are calculated as 2^-ΔΔ^*^CT^*. Three independent experiments were performed.

## Results

### *Cronobacter* Species Produce a Large Amount of Exopolysaccharide When Grown in M9

**FIGURE 1:**
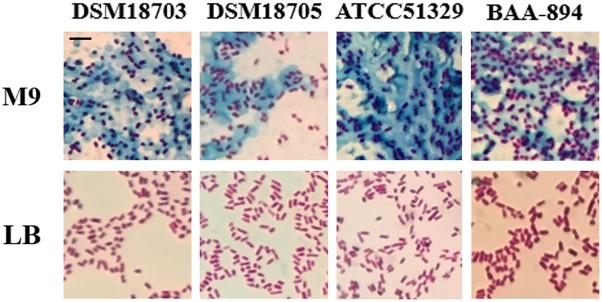
Light microscopic observation of four *Cronobacter* species grown on solid LB or M9 media and stained with mordant. Scale bar, 10 μm.

When four *Cronobacter* species (*C. turicensis* DSM18703, *C. dublinensis* DSM18705, *C. muytjensii* ATCC51329, and *C. sakazakii* BAA-894) were grown on solid M9 medium, they all formed mucoid colonies; however, when they were grown on solid LB medium, no mucoid colonies were observed. Mucoid colonies are usually associated with excessive production of exopolysaccharide, which can be confirmed by tannin mordant staining. Therefore, colonies of *Cronobacter* species DSM18703, DSM18705, ATCC51329, and BAA-894 grown on solid LB or M9 media were stained with tannin mordant and observed under microscopy (**Figure 1**). Large amounts of bluish substance were observed around the cells of *Cronobacter* species DSM18703, DSM18705, ATCC51329, or BAA-894 grown on M9, but no bluish substance was observed around the cells grown on LB (**Figure 1**). The LB medium contains tryptone, a pancreatic digest of casein which contains various amino acids, while M9 medium contains no amino acids. The results demonstrate that *Cronobacter* species could produce a large amount of exopolysaccharide when grown in the amino acid deficient medium M9 but not LB, suggesting the production of exopolysaccharide might be related to the availability of amino acids in the medium.

### Colanic Acid Is Produced in *C. sakazakii* BAA-894 Grown in M9 and the Production Is Regulated by the Rcs Phosphorelay System

**FIGURE 2:**
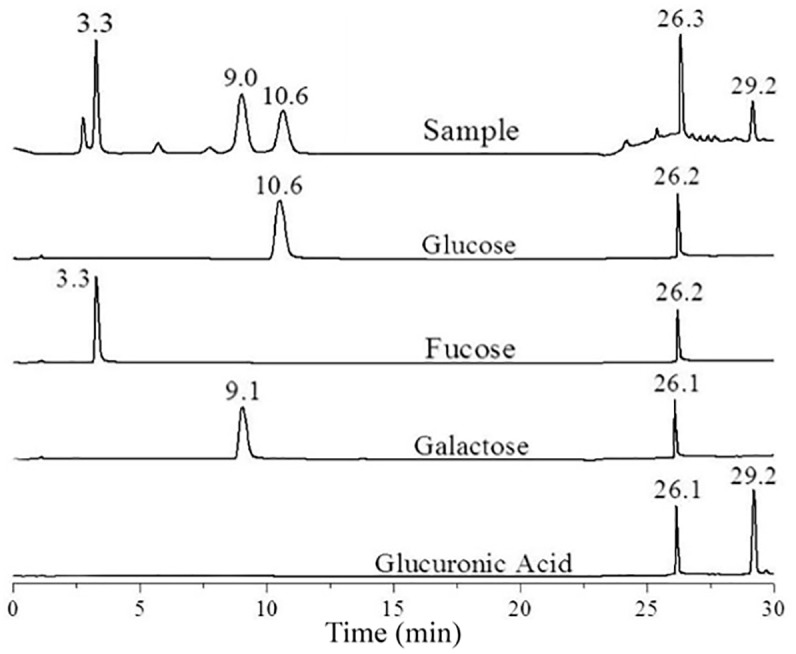
High-performance liquid chromatography analysis of the hydrolyzed exopolysaccharide isolated from *Cronobacter sakazakii* BAA-894 cells grown in liquid M9 medium.

Several types of exopolysaccharides can be produced in *C. sakazakii* (Ogrodzki and Forsythe, 2017). To determine the type of the exopolysaccharide produced by *C. sakazakii* grown in M9, *C. sakazakii* BAA-894 cells grown in liquid M9 medium, exopolysaccharide was collected from the cell culture, purified, hydrolyzed, and analyzed by HPLC (**Figure 2**). In the chromatogram, five peaks with different retention times (3.3, 9.0, 10.6, 26.3, and 29.2 min) were observed. Under the same analysis condition, fucose, glucose, galactose, and glucuronic acid yielded peaks at 3.3, 9.1, 10.6, and 29.2 min, respectively. Fucose, glucose, galactose, and glucuronic acid are the major components of *E. coli* CA (Sutherland, 1969). This analysis demonstrates that the major exopolysaccharide secreted by BAA-894 grown in M9 is CA, and *C. sakazakii* and *E. coli* share the similar CA structure. The strong peak around 26.2 min observed in all spectra was caused by the gradient change of the mobile phase during analysis (**Figure 2**).

**FIGURE 3:**
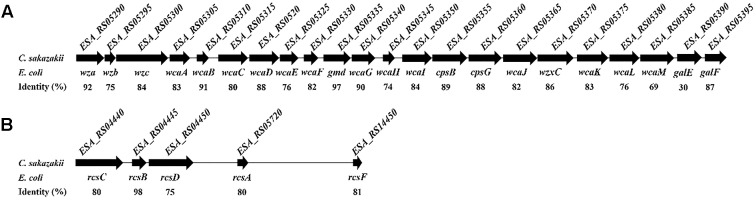
Homolog genes of *Escherichia coli wca* cluster **(A)** and Rcs phosphorelay system **(B)** exist in *C. sakazakii* BAA-894.

**FIGURE 4:**
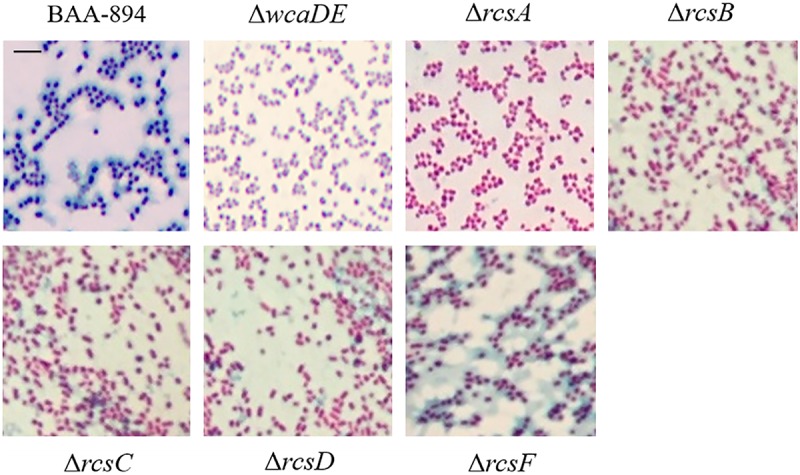
Light microscopic observation of wild type BAA-894 and various *C. sakazakii* mutants grown on M9 solid media and stained with mordant. Scale bar, 10 μm.

In *E. coli*, the *wca* cluster encodes the enzymes for CA biosynthesis (Stevenson et al., 1996), and its expression is regulated by the Rcs phosphorelay system (Majdalani and Gottesman, 2005). All the homolog genes relevant to CA biosynthesis and the Rcs phosphorelay system also exist in *C. sakazakii* BAA-894 (**Figure 3**). Two variants of the CA synthesis gene cluster (CA1 and CA2) are found in *C. sakazakii* isolates, and the ones containing CA2 are associated with neonatal meningitis and necrotizing enterocolitis (Ogrodzki and Forsythe, 2015). The CA1 and CA2 differ with the absence of *galE* in CA2. *C. sakazakii* BAA-894 does contain *galE* in its CA cluster (**Figure 3A**), but its identity with *E. coli galE* is only 30%, while the other genes in the cluster show at least 69% identity with their *E. coli* counterparts. To confirm if the exopolysaccharide secreted by *C. sakazakii* BAA-894 cells is CA, two contiguous genes *ESA_RS05320* (homolog of *E. coli wcaD*) and *ESA_RS05325* (homolog of *E. coli wcaE*) in the chromosome of BAA-894 were deleted, resulting in the mutant strain Δ*wcaDE*. WcaD is the CA polymerase and plays an important role in the CA biosynthesis. When Δ*wcaDE* grown on solid M9 medium and stained with tannin mordant, blue mucoid exopolysaccharide was not observed around the cells (**Figure 4**), suggesting that the exopolysaccharide secreted by *C. sakazakii* BAA-894 cells grown on M9 is CA. *E. coli* Rcs phosphorelay system contains proteins encoded by the genes *rcsA*, *rcsB*, *rcsC*, *rcsD*, and *rcsF. ESA_RS05720* (homolog of *rcsA*), *ESA_RS04445* (homolog of *rcsB*), *ESA_RS04440* (homolog of *rcsC*), *ESA_RS04450* (homolog of *rcsD*), or *ESA_RS14450* (homolog of *rcsF*) were individually deleted in *C. sakazakii* BAA-894, resulting in the mutant strains Δ*rcsA*, Δ*rcsB*, Δ*rcsC*, Δ*rcsD*, and Δ*rcsF*, respectively. When grown on solid M9 medium and stained with tannin mordant, no blueish mucoid CA was observed around cells of Δ*rcsA*, Δ*rcsB*, Δ*rcsC*, or Δ*rcsD* (**Figure 4**), suggesting that CA production in *C. sakazakii* cells is regulated by Rcs phosphorelay system. Interestingly, a small amount of bluish mucoid CA was observed around cells of Δ*rcsF* (**Figure 4**). Considering RcsF is the sensor of Rcs system, the results suggest that the production of CA in *C. sakazakii* cells is not 100% controlled by the Rcs system or there might be another protein which can function as RcsF.

### Colanic Acid Production in *C. sakazakii* BAA-894 Is Dependent on the Availability of Amino Acids

**FIGURE 5:**
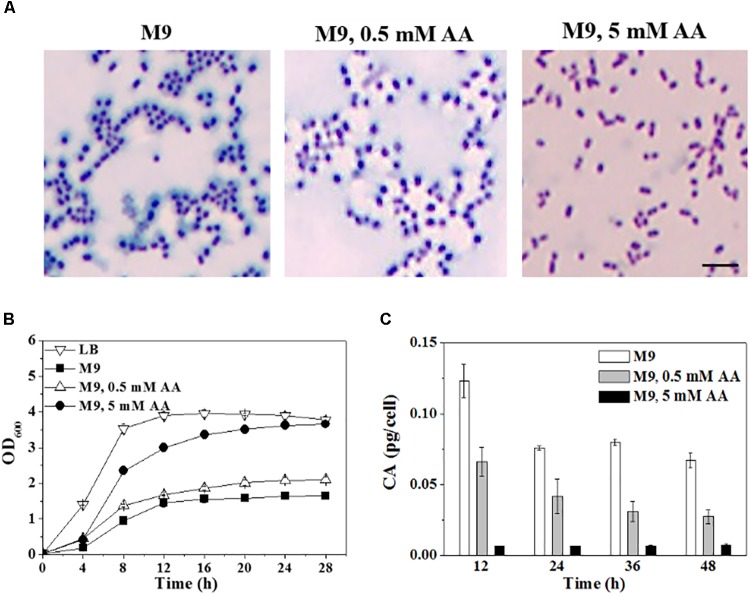
**(A)** Light microscopic observation of BAA-894 grown on media of M9, M9 containing 0.5 mM or 5 mM amino acids (AA) and stained with mordant. Scale bar, 10 μm. **(B)** Growth comparison of *C. sakazakii* BAA-894 cells grown in media of M9, M9 containing 0.5 mM or 5 mM AA. **(C)** Quantification of colanic acid produced in *C. sakazakii* BAA-894 grown in different liquid media.

Since amino acids are not contained in M9, *C. sakazakii* BAA-894 cells were also grown on solid M9 medium supplemented with 0.5 mM or 5 mM of 15 important amino acids (Asp, Glu, Ser, His, Gly, Thr, Arg, Ala, Cys, Val, Met, Phe, Ile, Leu, and Lys), and the colonies were stained with tannin mordant and observed under microscopy (**Figure 5A**). Compared to BAA-894 cells grown on M9, much less bluish substance was observed around BAA-894 cells grown on M9 supplemented with 0.5 mM amino acids, but no bluish substance was observed around BAA-894 cells grown on M9 supplemented with 5 mM amino acids (**Figure 5A**). This indicates that CA production in *C. sakazakii* BAA-894 is dependent on the availability of amino acids.

To confirm whether amino acid availability is the major factor for the slow growth of bacteria in M9, *C. sakazakii* BAA-894 cells were grown in liquid M9 supplemented with the 15 amino acids. When 0.5 mM amino acids were added in M9, the cell growth slightly improved (**Figure 5B**). When 5 mM amino acids were added in M9, the cell growth significantly improved, and its maximum OD_600_ reached 3.6, similar to the cells grown in LB (**Figure 5B**). The results suggest that *C. sakazakii* can quickly adapt to the environment without amino acids, and efficiently consume the amino acids when available.

Colanic acid production were also analyzed when *C. sakazakii* BAA-894 grown in liquid media of M9 and M9 supplemented with 0.5 mM or 5 mM amino acids (**Figure 5C**). Samples were collected at 12, 24, 36, and 48 h, and the CA levels were determined. A significant amount of CA was detected in cells grown in M9, and a small amount of CA was detected in cells grown in M9 supplemented with 0.5 mM amino acids; however, only a negligible amount of CA was detected in cells grown in M9 supplemented with 5 mM amino acids. Colanic acid production is closely related to the cell growth of bacteria. The worst the cells grow; the more CA be produced.

**FIGURE 6:**
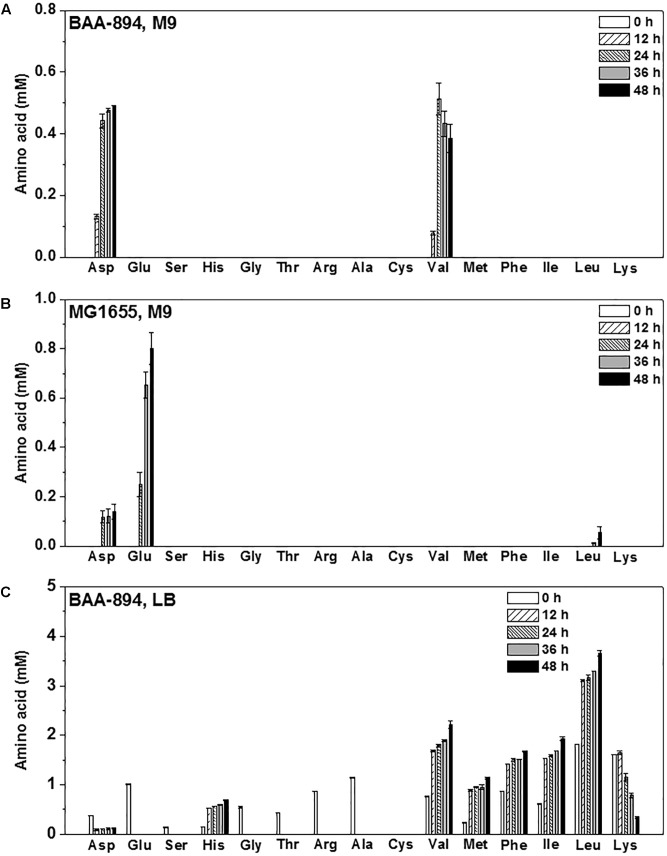
Levels of 15 amino acids in the culture of *C. sakazakii* BAA-894 grown in M9 **(A)**, *E. coli* MG1655 grown in M9 **(B)**, and BAA-894 grown in LB **(C)** medium at different time points.

Since amino acid availability is important for the cell growth and CA production in *C. sakazakii*, the levels of the 15 important amino acids produced by *E. coli* MG1655 grown in M9, *C. sakazakii* BAA-894 grown in M9 and LB were analyzed. M9 medium contains no amino acids, *C. sakazakii* BAA-894 grown in M9 medium accumulated a large amount of Asp and Val (**Figure 6A**), while *E. coli* MG1655 grown in M9 accumulated a large amount of Glu (**Figure 6B**). This indicates that the response mechanism of *C. sakazakii* and *E. coli* to amino acid deficiency might be quite different. The LB medium contains all the 15 amino acids, except for Cys; the top four high content amino acids (about 1–2 mM) are Leu, Lys, Glu, and Ala. When grown in LB, *C. sakazakii* BAA-894 consumed all Glu, Ser, Gly, Thr, Arg, and Ala in 12 h, but accumulated Asp, His, Val, Met, Phe, Ile, Leu, and Lys for 48 h (**Figure 6C**). This indicates that priority for amino acid synthesis and accumulation in *C. sakazakii* depend on the growth condition.

### Antibiotic Resistance of *Cronobacter sakazakii* BAA-894 Depends on the Growth Medium

**FIGURE 7:**
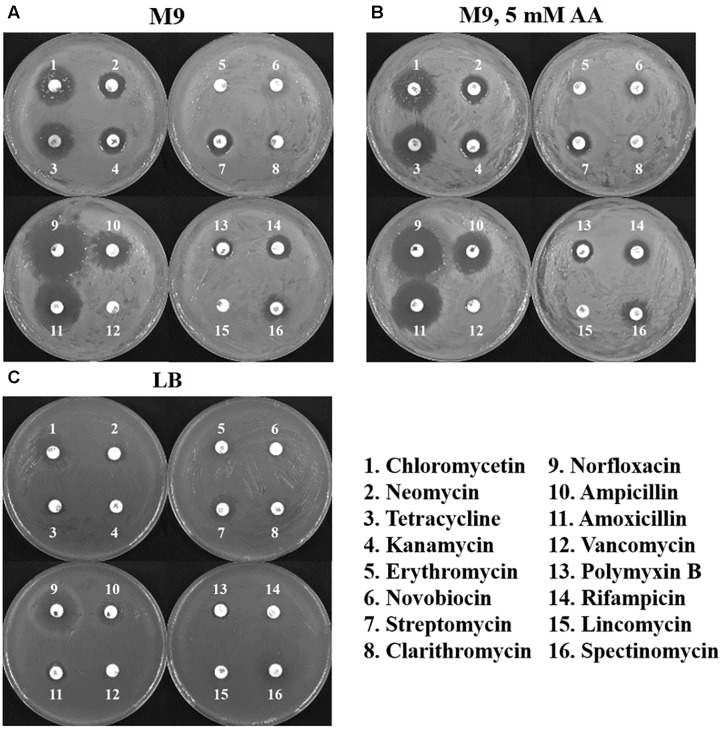
Disk diffusion assays of *C. sakazakii* BAA-894 cells grown in the medium of M9 **(A)**, M9 containing 5 mM AA **(B)**, and LB **(C)**.

Antibiotic therapy is the common way to prevent the *Cronobacter* infection in humans. Therefore, the resistance of *C. sakazakii* BAA-894 grown in different media to 16 antibiotics was evaluated with an antibiotic disk diffusion assay (**Figure 7**). *C. sakazakii* BAA-894 grown in M9 displayed sensitivity to at least 12 antibiotics. Interestingly, the resistance patterns for the 16 antibiotics for *C. sakazakii* BAA-894 grown in M9 (**Figure 7A**) are the same for *C. sakazakii* grown in M9 supplemented with 5 mM AA (**Figure 7B**), suggesting that amino acid availability does not affect the antibiotic resistance of *C. sakazakii* grown in M9. *C. sakazakii* BAA-894 grown in LB displayed resistance to at least 10 antibiotics (**Figure 7C**). *C. sakazakii* BAA-894 cells were very sensitive to norfloxacin no matter they were grown on LB or M9. *C. sakazakii* BAA-894 cells grown on M9 were also very sensitive to chloromycetin, tetracycline, ampicillin and amoxicillin. Norfloxacin can interfere with DNA synthesis by inhibiting bacterial DNA gyrase. Ampicillin and amoxicillin can inhibit the synthesis of the bacterial cell wall. Tetracycline can bind to the 30S subunit of ribosome, thereby inhibiting protein synthesis in bacteria. Generally, *C. sakazakii* BAA-894 was less sensitive to antibiotics when grown in LB than in M9 or M9 supplemented with 5 mM AA, possibly because its growth rate was better in LB than in M9 (**Figure 5B**). The bacterial cell fitness might play an important role for antibiotic resistance.

### When *Cronobacter sakazakii* BAA-894 Was Grown in M9, Genes Relevant to Exopolysaccharide Biosynthesis Were Significantly Up-Regulated, but Genes Relevant to Flagellum Biosynthesis and Chemotaxis Were Significantly Down-Regulated

To investigate the response of *C. sakazakii* BAA-894 to amino acid deficiency, total RNAs isolated from BAA-894 grown in LB or M9 media were analyzed. The total number of detected genes from BAA-894 grown in M9 and LB media were 3998 and 3981, respectively. Comparing the BAA-894 cells grown in LB, 2339 genes were up-regulated and 1617 genes were down-regulated in BAA-894 cells grown in M9, and the number of significantly modulated genes was 1263 (785 up-regulated and 478 down-regulated) (**Table 3** and **Supplementary Table S1**).

**Table 3:** List of the significantly regulated genes (| log_2_R|≥ 5) in *Cronobacter sakazakii* BAA-894 cells grown in M9 medium, using the same strain grown in LB medium as the control.

Genes		log_2_^R^	Function
		
BAA-894	MG1655		
ESA_RS12970	*ykgM*	8.13	50S ribosomal protein L31
ESA_RS19300	*zinT*	7.68	Metal-binding protein
ESA_RS03665	*rbsC*	6.97	ABC transporter permease
ESA_RS13555	*tauA*	6.97	Taurine ABC transporter substrate-binding protein
ESA_RS16935	*thiE*	6.88	Thiamine phosphate synthase
ESA_RS02415	*cysD*	6.83	Sulfate adenylyltransferase subunit 2
ESA_RS13090	*amtB*	6.79	Ammonium transporter
ESA_RS02410	*cysG*	6.79	Siroheme synthase
ESA_RS02425	*cysC*	6.78	Adenylyl-sulfate kinase
ESA_RS07295	*yciW*	6.71	Oxidoreductase
ESA_RS20880	N/A	6.69	Unknown
ESA_RS20875	N/A	6.68	Unknown
ESA_RS02420	*cysN*	6.6	Sulfate adenylyltransferase
ESA_RS03740	*cysA*	6.52	Sulfate ABC transporter ATP-binding protein
ESA_RS03675	N/A	6.50	Hypothetical protein
ESA_RS11400	*artJ*	6.46	Arginine ABC transporter substrate-binding protein
ESA_RS13095	*glnK*	6.45	Nitrogen regulatory protein P-II 2
ESA_RS03670	N/A	6.43	Sugar ABC transporter substrate-binding protein
ESA_RS04175	*yfcG*	6.4	Thiol:disulfide oxidoreductase
ESA_RS16950	*thiG*	6.30	Thiazole synthase
ESA_RS05615	*cbl*	6.30	CysB family transcriptional regulator
ESA_RS03660	N/A	6.30	Sugar ABC transporter ATP-binding protein
ESA_RS16940	*thiF*	6.15	Molybdopterin biosynthesis protein
ESA_RS03730	*cysU*	6.15	Sulfate/thiosulfate transporter subunit
ESA_RS20760	N/A	6.08	Unknown
ESA_RS03725	*cysP*	6.07	Thiosulfate transporter subunit
ESA_RS09560	*flu*	5.98	Hypothetical protein
ESA_RS16930	*thiC*	5.95	Phosphomethylpyrimidine synthase
ESA_RS19050	*sbp*	5.94	Sulfate transporter subunit
ESA_RS19460	*yhjE*	5.89	MFS transporter
ESA_RS03735	*cysW*	5.79	Sulfate/thiosulfate transporter permease subunit
ESA_RS16955	*thiH*	5.70	2-iminoacetate synthase
ESA_RS12640	N/A	5.62	Phage terminase large subunit
ESA_RS21110	N/A	5.54	Unknown
ESA_RS19775	*livK*	5.48	Leucine transporter subunit
ESA_RS13550	*tauB*	5.44	Taurine transport system ATP-binding protein
ESA_RS09565	N/A	5.28	Autotransporter strand-loop-strand *O*-heptosyltransferase
ESA_RS19785	*livM*	5.20	Branched-chain amino acid ABC transporter permease
ESA_RS03620	*entA*	5.19	2,3-dihydro-2,3-dihydroxybenzoate dehydrogenase
ESA_RS12560	*entF*	5.07	Enterobactin synthase subunit F
ESA_RS05610	*nac*	5.04	LysR family transcriptional regulator
ESA_RS08415	*bcsA*	5.02	Cellulose synthase
ESA_RS13320	*tsx*	-5.00	Nucleoside-specific channel-forming protein
ESA_RS01515	*yjiY*	-5.16	Carbon starvation protein A
ESA_RS13480	*yaiZ*	-5.42	Membrane protein
ESA_RS02285	*sdaB*	-5.51	L-serine ammonia-lyase
ESA_RS17695	*lldP*	-5.55	L-lactate permease
ESA_RS04410	*glpQ*	-5.83	Glycerophosphoryl diester phosphodiesterase
ESA_RS04990	*mglC*	-5.98	Galactoside ABC transporter permease
ESA_RS04980	*mglB*	-5.99	Galactose ABC transporter substrate-binding protein
ESA_RS04985	*mglA*	-6.05	Galactose/methyl galactoside ABC transporter ATP-binding protein
ESA_RS04405	*uhpC*	-6.65	MFS transporter
ESA_RS17675	*glpK*	-6.85	Glycerol kinase
ESA_RS01185	*treC*	-7.35	Glucohydrolase
ESA_RS17670	*glpF*	-7.40	Aquaporin
ESA_RS01190	*treB*	-8.34	PTS sucrose IIB component/PTS sucrose IIC component


**FIGURE 8:**
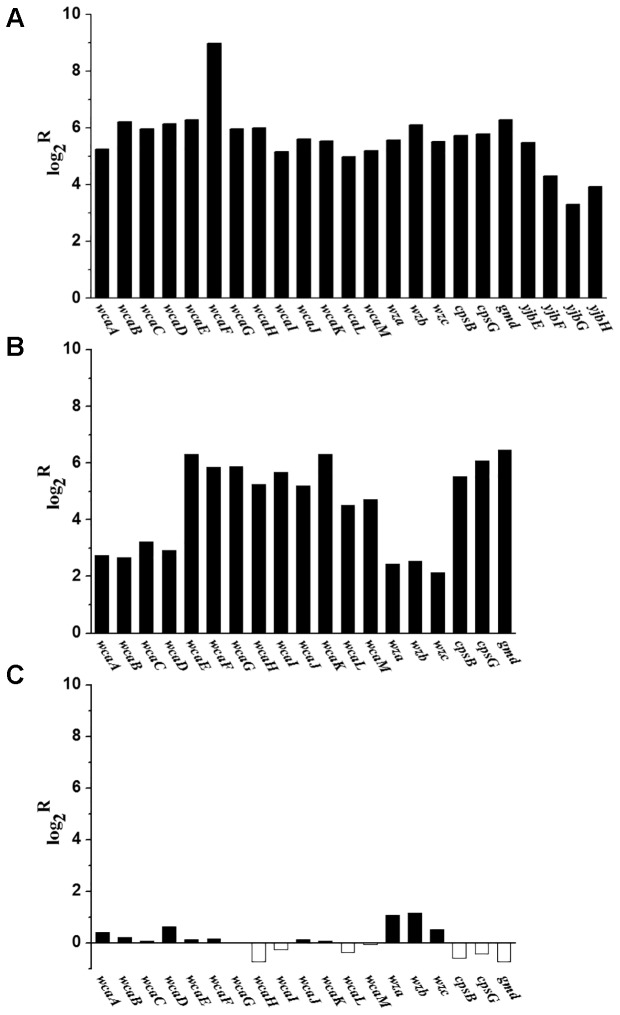
**(A)** Transcriptomic analysis showed that most genes involved in exopolysaccharide biosynthesis were up-regulated in *C. sakazakii* BAA-894 grown in M9 medium, using the ones grown in LB as the control. **(B)** RT-PCR analysis for transcriptional levels of some key genes involved in exopolysaccharide biosynthesis in *C. sakazakii* BAA-894 grown in M9 medium, using the ones grown in LB as the control. **(C)** RT-PCR analysis for transcriptional levels of some key genes involved in exopolysaccharide biosynthesis in *C. sakazakii* BAA-894 grown in M9 medium, using the ones grown in M9 with 5 mM amino acids as the control.

**FIGURE 9:**
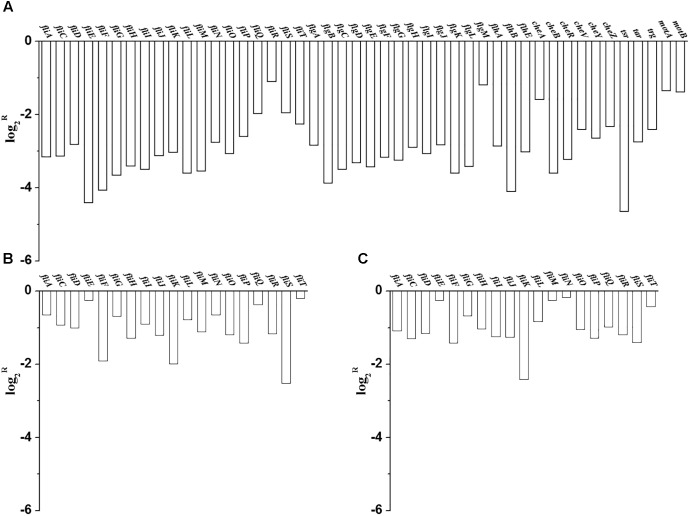
**(A)** Transcriptomic analysis showed that most genes involved in flagellum formation and chemotaxis were down-regulated in *C. sakazakii* BAA-894 grown in M9 medium. **(B)**. RT-PCR analysis for transcriptional levels of some key genes involved in flagellum formation in *C. sakazakii* BAA-894 grown in M9 medium, using the ones grown in LB as the control. **(C)** RT-PCR analysis for transcriptional levels of some key genes involved in flagellum formation in *C. sakazakii* BAA-894 grown in M9 medium, using the ones grown in M9 with 5 mM amino acids as the control.

**FIGURE 10:**
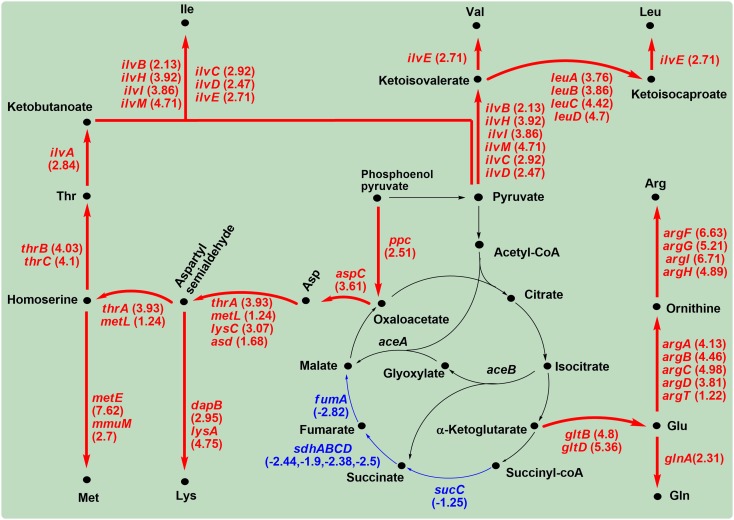
Significantly modulated genes involved in amino acid regulation in *C. sakazakii* BAA-894 grown in M9 medium. The up-regulated genes are shown in red, and the down-regulated genes shown in blue.

**The homologous BAA-894 genes were identified through BLAST, using the corresponding Escherichia coli MG1655 gene as a bait. N/A, no homolog gene was found in MG1655. The genes shown in **Figures 8–10** are not included in this table*.*

When grown in M9 medium, the transcriptional levels of 19 homolog genes involved in the biosynthesis of CA in *E. coli* were significantly up-regulated in *C. sakazakii* BAA-894 (**Figure 8A**). These transcriptomic analysis results were further confirmed by using RT-PCR analysis (**Figure 8B**). This suggests that the transcriptomic analysis used in this study is reliable. Interestingly, RT-PCR analysis showed that the transcriptional levels of some key genes involved in exopolysaccharide biosynthesis in *C. sakazakii* BAA-894 grown in M9 medium were similar to those grown in M9 with 5 mM amino acids (**Figure 8C**). The *yjbEFGH* operon is involved in the production of another exopolysaccharide in *E. coli* (Ferrieres et al., 2007). When grown in M9 medium, the transcriptional levels of the four homolog genes in the *yjbEFGH* operon were also significantly up-regulated in *C. sakazakii* BAA-894 (**Figure 8A**). The results are consistent with the observation of exopolysaccharide around cells of *C. sakazakii* BAA-894 grown in M9 as shown in **Figure 1**.

Flagellum is an accessory structure that protrudes from the bacterial cells, its primary role is locomotion, but it also functions as a sensory organelle to sense chemicals outside the cell (Silflow and Lefebvre, 2001; Bardy et al., 2003; Wang et al., 2005). When grown in M9 medium, the transcriptional levels of 35 genes relevant to flagellar biosynthesis and 11 genes relevant to chemotaxes were significantly down-regulated in *C. sakazakii* BAA-894 (**Figure 9A**). Similar transcriptional levels of some key genes relevant to flagellar biosynthesis were also observed by using RT-PCR analysis (**Figure 9B**). This suggests that the transcriptomic analysis used in this study is reliable. RT-PCR analysis also showed that the transcriptional levels of some key genes relevant to flagellar biosynthesis in *C. sakazakii* BAA-894 grown in M9 medium were down-regulated when compared to those grown in M9 with 5 mM amino acids (**Figure 9C**). This suggests that flagella might not be synthesized in *C. sakazakii* grown in M9 medium.

### Many Genes Relevant to the Biosynthesis of Various Amino Acids Were Significantly Regulated in *Cronobacter sakazakii* BAA-894 Grown in M9 Medium

When *C. sakazakii* BAA-894 cells were grown in M9 medium, genes *sucC*, *sdhA*, *sdhB*, *sdhC*, *sdhD*, and *fumA* were down-regulated, suggesting that the TCA cycle was weakened (**Figure 10**). The carbon flowed out from oxaloacetate, α-ketoglutarate and pyruvate to increase the production of various amino acids (**Figure 10**).

The up-regulated *ppc* and *aspC* directed the carbon flow from phosphoenol pyruvate to Asp. This is consistent with the high accumulation of Asp in *C. sakazakii* grown in M9 medium (**Figure 6A**). The up-regulated *thrA*, *metL*, *lysC*, *asd*, *thrB*, and *thrC* further convert Asp to Thr (Dong et al., 2011). Ile biosynthesis from Thr is a five-step pathway that shares its last four steps with the Val biosynthesis. The first step is catalyzed by IlvA; the second step can be catalyzed by each of the three enzyme pairs, IlvG-1/IlvM, IlvI/IlvH, and IlvB/IlvN; the last three steps are catalyzed by IlvC, IlvD, and IlvE, respectively. The key genes (*ilvA*, *ilvB*, *ilvC*, *ilvD*, *ilvE*, *ilvH*, *ilvI*, *ilvM*) encoding the enzymes for the five-step reactions were significantly up-regulated in *C. sakazakii* BAA-894 grown in M9 (**Figure 10**). Leu biosynthesis involves a five-step process starting from ketoisovalerate, and is catalyzed by LeuA, LeuB, LeuC, LeuD, and IlvE, respectively. The genes (*leuA*, *leuB*, *leuC*, *leuD*, *ilvE*) encoding these enzymes were also significantly up-regulated in *C. sakazakii* BAA-894 grown in M9 (**Figure 10**). LeuO is a Leu transcriptional activator. The ABC transporter complex LivFGHMJ can transport Leu, Ile, or Val, while the complex LivFGHMK is specific for transporting Leu. The genes (*livF*, *livG*, *livH*, *livJ*, *livK*, *livM*, and *leuO*) encoding these enzymes were significantly up-regulated in *C. sakazakii* BAA-894 grown in M9 medium (**Table 3**). The data suggest that the biosynthesis of Leu, Ile, and Val were enhanced in *C. sakazakii* in response to an extremely low concentration of amino acids in M9 medium. The biosynthesis of Lys and Met might also be enhanced, considering they also use Asp as the precursor. LysA catalyzes the last reaction for the biosynthesis of Lys; the transcriptional level of *lysA* was significantly up-regulated (Log_2_^R^ = 4.75) in *C. sakazakii* BAA-894 grown in M9 medium, and another key gene *dapB* in the Lys biosynthetic pathway was also up-regulated. *MetE* and *MmuM* catalyze the last reaction for the biosynthesis of Met; the transcriptional level of *metE* (Log_2_^R^ = 7.62) and *mmuM* (Log_2_^R^ = 2.7) was significantly up-regulated in *C. sakazakii* BAA-894 grown in M9 medium (**Figure 10**).

In *E. coli*, Glu provides nitrogen for other amino acids such as Asp, His, and Arg (Reitzer, 2003). Glu can also be synthesized from α-ketoglutarate by Glu synthase composed of GltB and GltD (Castaño et al., 1992). GlnA catalyzes the reaction of Glu and ammonia to generate glutamine Gln. When *C. sakazakii* BAA-894 was grown in M9 medium, the transcriptional levels of *glnA*, *gltB*, and *gltD* were significantly up-regulated (**Figure 10**). Glu can also be converted into L-ornithine by enzymes ArgA, ArgB, ArgC, ArgD, and ArgT, and L-ornithine can be further converted to Arg by enzymes ArgF, ArgI, ArgG, and ArgH. When *C. sakazakii* BAA-894 was grown in M9 medium, the transcriptional levels of *argA*, *argB*, *argC*, *argD*, *argF*, *argI*, *argG*, *argH*, and *argT* were significantly up-regulated (**Figure 10**).

TrpA, TrpD, and TrpE are key enzymes in the biosynthetic pathway of L-tryptophan. When grown in M9 medium, the transcriptional levels of *trpA*, *trpD*, and *trpE* were significantly up-regulated in *C. sakazakii* BAA-894 (**Table 3**). His biosynthesis is carried out by enzymes HisA, HisB, HisC, HisD, HisF, HisG, HisH, and HisI. HisFH catalyzes the fifth step of His biosynthesis. When grown in M9 medium, the transcriptional levels of *hisA*, *hisB*, *hisC*, *hisD*, *hisG*, *hisH*, and *hisI* were significantly up-regulated in *C. sakazakii* BAA-894 (**Table 3**). These data suggest that the L-tryptophan and His biosynthesis were enhanced in *C. sakazakii* grown in M9.

## Discussion

Bacteria can survive in various environments by modulating their intracellular metabolism and coordinating uptake of the primary substrates for biomass production (Doucette et al., 2011). The genes relevant to flagellum formation and chemotaxis were significantly down-regulated in *C. sakazakii* BAA-894 grown in the amino acid deficient medium M9 (**Figure 9**). The amino acid content is not the only variable in M9 and LB; another variable is glucose which is contained in M9 medium but not in LB. Glucose represses flagellar synthesis via cyclic-AMP, and the repression of flagellar genes in M9 can be attributed to glucose, and not differences in amino acid content. The lower expression of genes *emrA* (log_2_^R^ = -1.43), *emrD* (log_2_^R^ = -1.84), *emrE* (log_2_^R^ = -1.57), and *emrR* (log_2_^R^ = -1.34) encoding the multidrug resistance pumps *C. sakazakii* BAA-894 grown in M9 might also be attributable to glucose, considering the increased antibiotic sensitivity of *C. sakazakii* BAA-894 grown in M9 does not change after supplemented with 5 mM amino acids (**Figure 7**).

Because of the amino acid deficiency and the high glucose concentration in M9, the ratio of intracellular carbon to nitrogen might be much higher in M9 than in LB; the redundant carbon source in *C. sakazakii* might be used for synthesizing exopolysaccharide (**Figure 1**) to protect cells against environmental stress. Based on the transcriptomic analysis, the genes relevant to CA biosynthesis were significantly up-regulated (**Figure 8**). At least four types of exopolysaccharides (CA, an exopolysaccharide produced by the *yjbEFGH* operon, K-antigen, and cellulose) can be produced in *C. sakazakii* (Ferrieres et al., 2007; Ogrodzki and Forsythe, 2017). When *C. sakazakii* BAA-894 cells were grown in M9, all 19 genes relevant to CA production and all 4 genes in the *yjbEFGH* operon were significantly up-regulated, whereas only one gene *kpsS* (Log_2_^R^ = 1.75) relevant to K-antigen production was slightly up-regulated and one gene *bcsA* (Log_2_^R^ = 5.02) relevant to cellulose production were significantly up-regulated. Our experiments demonstrate that CA is the major exopolysaccharide produced by *C. sakazakii* grown in M9, its production is dependent on the availability of amino acids and is regulated by the Rcs phosphorelay system; the detailed mechanism remains to be determined.

Amino acids are the most important nitrogen source for bacteria, their availability can lead to a coordinated regulation of metabolism. In *E. coli*, nitrogen regulation two-component system NtrB and NtrC are encoded by *glnL* and *glnG*, respectively (Zimmer et al., 2000), and nitrogen starvation results in the phosphorylation of the response regulator NtrC and the activation of RelA (Kuroda et al., 1999). RelA is responsible for synthesizing the alarmone ppGpp which causes a massive re-programming of the transcriptional profile known as the stringent response (Rombel et al., 1998; Wendrich et al., 2002; Shyp et al., 2012; Schumacher et al., 2013; Brown et al., 2014). In *E. coli*, the nitrogen stress response allows cells to rapidly sense nitrogen limitation, scavenge for alternative nitrogen sources through the transcriptional activation of transport systems and catabolic and biosynthetic operons, and adapt to nitrogen limitation. However, in *C. sakazakii* BAA-894 when grown in M9, though *glnG* and *glnL* were significantly up-regulated, the transcriptional level of *relA* did not change, suggesting that the nitrogen stress response did not occur in *C. sakazakii* cells. In fact, M9 medium contains 0.1% NH_4_Cl (18 mM), although it is amino acid deficient, it should be nitrogen rich. In *E. coli*, the phosphatase and kinase activities of NtrB are regulated by GlnB and GlnK; GlnK is tightly regulated under nitrogen-rich conditions, but expressed during ammonium starvation (Gosztolai et al., 2017). In *C. sakazakii* grown in M9, *glnK* (Log_2_^R^ = 6.45) and *amtB* (Log_2_^R^ = 6.79) encoding an ammonium transporter AmtB were significantly up-regulated. This suggests that the response to amino acid deficiency in *C. sakazakii* is different from that in *E. coli*. GlnK provides a functional link between nitrogen and carbon metabolisms (van Heeswijk et al., 2013; Gosztolai et al., 2017), therefore, the overexpression of AmtB and GlnK might balance the ratio of carbon to nitrogen for the benefit of *C. sakazakii* under amino acid deficiency.

Since M9 medium contains no amino acids, *C. sakazakii* grown in M9 must synthesize the necessary amino acids for cell growth. The transcriptional levels of most genes related to biosynthesis of various amino acids were significantly regulated in *C. sakazakii* BAA-894 grown in M9 (**Figure 10**). This indicates that balancing the amount of various amino acids is the major task for *C. sakazakii* to grow under the condition of amino acid deficiency. With respect to the amino acid accumulation in the medium, Glu and Asp accumulation in *E. coli* makes sense; because the concentration of Glu is high, and reversible transamination elevates Asp. The surprising result is that Asp and Val are high in *C. sakazakii*; Asp is high possibly because of high phosphoenol pyruvate, and Val is high possibly because of high pyruvate (**Figure 10**). These differences suggest that the response mechanism to amino acid deficiency in *C. sakazakii* is quite different from that in *E. coli*.

## Conclusion

*Cronobacter* species can cause necrotizing enterocolitis and meningitis. Their responses to extreme growth conditions could provide important information on their infection mechanism. This study investigated the response of *C. sakazakii* to amino acid deficiency. *C. sakazakii* produced CA when grown in the amino acid deficient M9 but not in the amino acid rich LB media; CA production is regulated by the Rcs phosphorelay system and depends on the availability of amino acids. Transcriptomes *C. sakazakii* BAA-894 grown in M9 or LB showed that 3956 genes were differentially expressed. When *C. sakazakii* BAA-894 was grown in M9, the genes relevant to the biosynthesis of CA were significantly up-regulated, but the genes relevant to the flagellum formation and chemotaxis were significantly down-regulated; most genes relevant to various amino acid biosynthesis were also significantly regulated. The results demonstrate that amino acid deficiency has a global impact on *C. sakazakii* cells. Since CA, flagella and chemotaxis are associated with pathogenesis of *C. sakazakii*, the data on *C. sakazakii* responses to amino acid deficiency might provide information for better control the infection.

## Author Contributions

SC, YL, and XW conceived and designed the experiments. SC, QZ, XT, and GR performed the experiments. SC and XW analyzed the data. XW and YL contributed reagents, materials, and analysis tools. SC and XW wrote the paper.

## Conflict of Interest Statement

The authors declare that the research was conducted in the absence of any commercial or financial relationships that could be construed as a potential conflict of interest.

## Footnotes

1 http://www.kegg.jp/kegg/kegg2.html

2 http://www.geneontology.org/

3 http://search.cpan.org/dist/GO-TermFinder/
