# Supplementary material for: The Global Response of Cronobacter sakazakii Cells to Amino Acid Deficiency
Source: Front Microbiol. 2018 Aug 14;9:1875. doi: 10.3389/fmicb.2018.01875 (PMC6102319; doi:10.3389/fmicb.2018.01875)
Supplement: Supplementary file 1 [file Table_1.docx]

**Table S1.** List of the genes that were significantly regulated (5≥|log_2_R|) in *C. sakazakii* BAA-894 grown in M9 medium, using the same strain grown in LB medium as a control. The homologous BAA-894 genes were identified through BLAST, using the corresponding MG1655 gene as a bait. N/A, no homologue gene is found in MG1655. The genes shown in Fig. 4 and Fig. 5 are not included here.

| Genes | | log_2_^R^ | Function |
| --- | --- | --- | --- |
| BAA-894 | MG1655 |  |  |
| ESA_RS19780 | *livH* | 4.93 | branched-chain amino acid ABC transporter permease |
| ESA_RS05370 | *wzxC* | 4.91 | lipopolysaccharide biosynthesis protein |
| ESA_RS03615 | *entB* | 4.89 | isochorismatase |
| ESA_RS03610 | *entE* | 4.88 | 2,3-dihydroxybenzoate-AMP ligase |
| ESA_RS05195 | *thiM* | 4.88 | hydroxyethylthiazole kinase |
| ESA_RS00500 | *yjcB* | 4.87 | membrane protein |
| ESA_RS19795 | *livF* | 4.87 | ABC transporter ATP-binding protein |
| ESA_RS19765 | *livK* | 4.86 | branched chain amino acid ABC transporter substrate-binding protein |
| ESA_RS16835 | *aapJ* | 4.85 | amino acid ABC transporter substrate-binding protein |
| ESA_RS06385 | *edd* | 4.80 | phosphogluconate dehydratase |
| ESA_RS17605 | *metF* | 4.80 | 5,10-methylenetetrahydrofolate reductase |
| ESA_RS11830 | *bioC* | 4.78 | malonyl-[acyl-carrier protein] O-methyltransferase BioC |
| ESA_RS03605 | *entC* | 4.76 | isochorismate synthase EntC |
| ESA_RS05720 | *rcsA* | 4.69 | helix-turn-helix transcriptional regulator |
| ESA_RS05200 | *thiD* | 4.68 | bifunctional hydroxymethylpyrimidine kinase/phosphomethylpyrimidine kinase |
| ESA_RS13545 | *tauC* | 4.66 | taurine ABC transporter permease |
| ESA_RS05490 | *hisC* | 4.65 | histidinol-phosphate aminotransferase |
| ESA_RS05495 | *hisD* | 4.60 | histidinol dehydrogenase |
| ESA_RS03625 | *entH* | 4.53 | proofreading thioesterase EntH |
| ESA_RS07480 | N/A | 4.51 | hypothetical protein |
| ESA_RS19790 | *livG* | 4.45 | ABC transporter ATP-binding protein |
| ESA_RS11835 | *bioF* | 4.44 | 8-amino-7-oxononanoate synthase |
| ESA_RS03920 | *alsS* | 4.39 | acetolactate synthase AlsS |
| ESA_RS05500 | *hisG* | 4.37 | ATP phosphoribosyltransferase |
| ESA_RS09820 | *ydjN* | 4.35 | L-cystine transporter |
| ESA_RS18510 | *asnA* | 4.30 | aspartate--ammonia ligase |
| ESA_RS12565 | *ybdZ* | 4.30 | MbtH family protein |
| ESA_RS07160 | *trpD* | 4.27 | bifunctional glutamine amidotransferase/anthranilate phosphoribosyltransferase |
| ESA_RS01890 | *ser* | 4.25 | D-3-phosphoglycerate dehydrogenase |
| ESA_RS11415 | *potI* | 4.24 | putrescine ABC transporter permease PotI |
| ESA_RS03655 | *ybdH* | 4.22 | oxidoreductase |
| ESA_RS02400 | *cysH* | 4.15 | phosphoadenosine phosphosulfate reductase |
| ESA_RS02395 | *cysI* | 4.13 | assimilatory sulfite reductase (NADPH) hemoprotein subunit |
| ESA_RS16060 | *pat)* | 4.12 | putrescine aminotransferase |
| ESA_RS21185 | N/A | 4.12 | Unknown |
| ESA_RS07165 | *trpE* | 4.10 | anthranilate synthase component I |
| ESA_RS11825 | *bioD* | 4.09 | dethiobiotin synthase |
| ESA_RS12575 | *fepA* | 4.05 | TonB-dependent siderophore receptor |
| ESA_RS16160 | *yhcF* | 4.01 | hypothetical protein |
| ESA_RS09425 | N/A | 4.01 | hypothetical protein |
| ESA_RS06910 | N/A | 3.99 | hypothetical protein |
| ESA_RS02535 | *glnH* | 3.97 | basic amino acid ABC transporter substrate-binding protein |
| ESA_RS08120 | N/A | 3.97 | allophanate hydrolase |
| ESA_RS21180 | N/A | 3.96 | Unknown |
| ESA_RS11420 | *potH* | 3.94 | putrescine ABC transporter permease |
| ESA_RS21175 | N/A | 3.93 | Unknown |
| ESA_RS06765 | *fhuA* | 3.92 | TonB-dependent siderophore receptor |
| ESA_RS15165 | *carB* | 3.90 | carbamoyl phosphate synthase large subunit |
| ESA_RS03915 | *ydbD* | 3.88 | alpha-acetolactate decarboxylase |
| ESA_RS08625 | *yncE* | 3.86 | hypothetical protein |
| ESA_RS03680 | *ubiB* | 3.85 | 5-methylthioribose kinase |
| ESA_RS06355 | *znuA* | 3.84 | zinc ABC transporter substrate-binding protein |
| ESA_RS21190 | N/A | 3.83 | Unknown |
| ESA_RS21355 | *asnC* | 3.80 | transcriptional activator of asnA; autorepressor |
| ESA_RS12570 | *fes* | 3.79 | enterochelin esterase |
| ESA_RS08335 | *tsr* | 3.73 | methyl-accepting chemotaxis protein |
| ESA_RS02390 | *cysJ* | 3.70 | assimilatory sulfite reductase (NADPH) flavoprotein subunit |
| ESA_RS07115 | *fepA* | 3.69 | TonB-dependent siderophore receptor |
| ESA_RS11425 | *potG* | 3.69 | polyamine ABC transporter ATP-binding protein |
| ESA_RS12685 | N/A | 3.69 | hypothetical protein |
| ESA_RS15170 | *carA* | 3.66 | carbamoyl-phosphate synthase small subunit |
| ESA_RS20160 | *nirD* | 3.65 | nitrite reductase small subunit |
| ESA_RS08895 | *dppB* | 3.64 | ABC transporter permease |
| ESA_RS18350 | N/A | 3.64 | AzlD domain-containing protein |
| ESA_RS06265 | N/A | 3.61 | aspartate aminotransferase |
| ESA_RS11840 | *bioB* | 3.57 | biotin synthase |
| ESA_RS13420 | *yaiA* | 3.57 | hypothetical protein |
| ESA_RS21170 | N/A | 3.55 | Unknown |
| ESA_RS10275 | *fhuE* | 3.53 | TonB-dependent siderophore receptor |
| ESA_RS02670 | *nrdF* | 3.52 | ribonucleotide-diphosphate reductase subunit beta |
| ESA_RS08090 | *bluR* | 3.51 | hypothetical protein |
| ESA_RS09775 | N/A | 3.50 | hypothetical protein |
| ESA_RS18340 | *pspE* | 3.47 | rhodanese |
| ESA_RS13635 | *dauA* | 3.46 | SulP family inorganic anion transporter |
| ESA_RS14255 | N/A | 3.43 | hypothetical protein |
| ESA_RS02540 | *yecS* | 3.42 | amino acid ABC transporter permease |
| ESA_RS08125 | *accC* | 3.42 | urea carboxylase |
| ESA_RS11845 | *bioA* | 3.41 | adenosylmethionine--8-amino-7-oxononanoate aminotransferase BioA |
| ESA_RS18345 | N/A | 3.41 | hypothetical protein |
| ESA_RS05530 | N/A | 3.41 | acyltransferase |
| ESA_RS05485 | *hisB* | 3.40 | bifunctional imidazole glycerol-phosphate dehydratase/histidinol phosphatase |
| ESA_RS14245 | N/A | 3.38 | hypothetical protein |
| ESA_RS18355 | *ygaZ* | 3.37 | branched-chain amino acid ABC transporter permease |
| ESA_RS21295 | N/A | 3.36 | Unknown |
| ESA_RS14155 | N/A | 3.34 | hypothetical protein |
| ESA_RS03925 | *ucpA* | 3.33 | acetoin reductase |
| ESA_RS06350 | *znuC* | 3.33 | zinc ABC transporter ATP-binding protein ZnuC |
| ESA_RS03770 | *cysK* | 3.32 | cysteine synthase A |
| ESA_RS14065 | *pfo* | 3.31 | hypothetical protein |
| ESA_RS02545 | *glnQ* | 3.29 | amino acid ABC transporter ATP-binding protein |
| ESA_RS13860 | N/A | 3.27 | Unknown |
| ESA_RS18360 | N/A | 3.26 | ribosomal-protein-S18p-alanine acetyltransferase |
| ESA_RS05670 | *yedJ* | 3.26 | phosphohydrolase |
| ESA_RS08305 | *ydeA* | 3.21 | sugar transporter |
| ESA_RS18335 | N/A | 3.20 | hypothetical protein |
| ESA_RS11850 | *yhbO* | 3.20 | type 1 glutamine amidotransferase domain-containing protein |
| ESA_RS12535 | *ybdO* | 3.19 | LysR family transcriptional regulator |
| ESA_RS05080 | *yehY* | 3.19 | osmoprotectant uptake system permease |
| ESA_RS03070 | *grcA* | 3.18 | autonomous glycyl radical cofactor GrcA |
| ESA_RS13805 | *dppA* | 3.18 | ABC transporter substrate-binding protein |
| ESA_RS05470 | N/A | 3.18 | Unknown |
| ESA_RS05480 | *hisH* | 3.15 | imidazole glycerol phosphate synthase subunit |
| ESA_RS13680 | *nlpA* | 3.15 | methionine ABC transporter substrate-binding protein |
| ESA_RS18365 | *bsmA* | 3.13 | protein BsmA |
| ESA_RS05475 | *hisA* | 3.10 | 1-(5-phosphoribosyl)-5-((5-phosphoribosylamino)methylideneamino)imidazole-4-carboxamide isomerase |
| ESA_RS05465 | *hisI* | 3.09 | bifunctional phosphoribosyl-AMP cyclohydrolase/phosphoribosyl-ATP diphosphatase |
| ESA_RS10510 | *yceI* | 3.03 | YceI family protein |
| ESA_RS14200 | N/A | 3.00 | hypothetical protein |
| ESA_RS07045 | *adhE* | 2.98 | bifunctional acetaldehyde-CoA/alcohol dehydrogenase |
| ESA_RS07485 | N/A | 2.98 | hypothetical protein |
| ESA_RS18015 | *rshE* | 2.96 | hypothetical protein |
| ESA_RS08945 | *pntB* | 2.96 | NAD(P)(+) transhydrogenase (Re/Si-specific) subunit beta |
| ESA_RS20165 | *nirB* | 2.95 | nitrite reductase large subunit |
| ESA_RS15175 | *dapB* | 2.95 | 4-hydroxy-tetrahydrodipicolinate reductase |
| ESA_RS09770 | *proP* | 2.95 | proline/betaine transporter |
| ESA_RS16675 | *yhcN* | 2.93 | membrane protein |
| ESA_RS13540 | *tauD* | 2.92 | taurine dioxygenase |
| ESA_RS10875 | *rutE* | 2.90 | nitroreductase family protein |
| ESA_RS07500 | N/A | 2.90 | hypothetical protein |
| ESA_RS16840 | *yhdX* | 2.86 | amino acid ABC transporter permease |
| ESA_RS16330 | *mtr* | 2.85 | tryptophan permease |
| ESA_RS08605 | N/A | 2.83 | sulfonate ABC transporter substrate-binding protein |
| ESA_RS12995 | N/A | 2.81 | Unknown |
| ESA_RS17195 | N/A | 2.79 | Unknown |
| ESA_RS21160 | N/A | 2.79 | Unknown |
| ESA_RS18740 | *xanP* | 2.77 | xanthine permease |
| ESA_RS03585 | *fepG* | 2.74 | iron-enterobactin transporter permease |
| ESA_RS07145 | *trpA* | 2.73 | tryptophan synthase subunit alpha |
| ESA_RS12990 | *metN* | 2.71 | ABC transporter |
| ESA_RS10060 | *CspBIJ* | 2.71 | cold-shock protein |
| ESA_RS12145 | *ybfA* | 2.71 | hypothetical protein |
| ESA_RS13560 | *mmuM* | 2.70 | homocysteine S-methyltransferase |
| ESA_RS12980 | N/A | 2.70 | 2-oxobutyrate oxidase |
| ESA_RS07150 | N/A | 2.70 | Unknown |
| ESA_RS03380 | *yfgJ* | 2.69 | hypothetical protein |
| ESA_RS14240 | N/A | 2.67 | recombinase |
| ESA_RS02675 | *nrdE* | 2.67 | ribonucleotide-diphosphate reductase |
| ESA_RS16845 | *yhdY* | 2.67 | amino acid ABC transporter permease |
| ESA_RS15045 | *thiS* | 2.66 | thiamine transporter substrate binding subunit |
| ESA_RS15655 | N/A | 2.65 | outer membrane autotransporter barrel domain-containing protein |
| ESA_RS02680 | *nrdI* | 2.62 | class Ib ribonucleoside-diphosphate reductase assembly flavoprotein NrdI |
| ESA_RS10825 | N/A | 2.60 | hypothetical protein |
| ESA_RS03595 | *entS* | 2.60 | MFS transporter |
| ESA_RS06395 | *purT* | 2.60 | phosphoribosylglycinamide formyltransferase 2 |
| ESA_RS08550 | *yahK* | 2.58 | hydroxyacid dehydrogenase |
| ESA_RS10055 | N/A | 2.58 | hypothetical protein |
| ESA_RS13630 | *yadF* | 2.58 | carbonic anhydrase |
| ESA_RS12270 | *asnB* | 2.55 | asparagine synthase B |
| ESA_RS11450 | *ybjM* | 2.55 | membrane protein |
| ESA_RS07560 | *ymfQ* | 2.55 | phage tail protein |
| ESA_RS06390 | *eda* | 2.54 | ketohydroxyglutarate aldolase |
| ESA_RS00275 | N/A | 2.53 | sodium transporter |
| ESA_RS17570 | *ppc* | 2.51 | phosphoenolpyruvate carboxylase |
| ESA_RS20155 | N/A | 2.51 | siroheme synthase |
| ESA_RS05060 | *mnaT* | 2.50 | N-acetyltransferase |
| ESA_RS06345 | *znuB* | 2.50 | zinc ABC transporter permease |
| ESA_RS17600 | *aer* | 2.49 | hypothetical protein |
| ESA_RS19865 | *gntK* | 2.49 | gluconokinase |
| ESA_RS19990 | *feoC* | 2.48 | ferrous iron transporter C |
| ESA_RS13465 | *mdtG* | 2.47 | MFS transporter |
| ESA_RS06785 | *yrdA* | 2.47 | hypothetical protein |
| ESA_RS19360 | *dppA* | 2.46 | ABC transporter substrate-binding protein |
| ESA_RS21155 | N/A | 2.46 | Unknown |
| ESA_RS03910 | *ynfL* | 2.45 | LysR family transcriptional regulator |
| ESA_RS03905 | *corA* | 2.43 | magnesium transporter CorA |
| ESA_RS16850 | *yhdZ* | 2.43 | amino acid ABC transporter ATP-binding protein |
| ESA_RS15050 | *thiP* | 2.43 | thiamine/thiamine pyrophosphate ABC transporter permease ThiP |
| ESA_RS08280 | *ydeE* | 2.43 | MFS transporter |
| ESA_RS08290 | *marB* | 2.42 | multiple antibiotic resistance regulatory periplasmic protein MarB |
| ESA_RS03580 | *fepC* | 2.42 | ferric enterobactin ABC transporter ATP-binding protein |
| ESA_RS12985 | N/A | 2.41 | metal ABC transporter substrate-binding protein |
| ESA_RS03175 | *purL* | 2.41 | phosphoribosylformylglycinamidine synthase |
| ESA_RS07155 | N/A | 2.41 | Unknown |
| ESA_RS08285 | *eamA* | 2.40 | O-acetylserine/cysteine exporter |
| ESA_RS19870 | *gntU* | 2.39 | gluconate transporter |
| ESA_RS18125 | N/A | 2.38 | Hcp1 family type VI secretion system effector |
| ESA_RS08950 | *pntA* | 2.36 | NAD(P)(+) transhydrogenase (Re/Si-specific) subunit alpha |
| ESA_RS05620 | *shiA* | 2.36 | MFS transporter |
| ESA_RS03085 | *nadB* | 2.35 | L-aspartate oxidase |
| ESA_RS08425 | *rob* | 2.33 | hypothetical protein |
| ESA_RS13565 | *mmuP* | 2.32 | S-methylmethionine permease |
| ESA_RS14835 | *aroP* | 2.31 | aromatic amino acid transporter AroP |
| ESA_RS18650 | *glnA* | 2.31 | glutamine synthetase |
| ESA_RS09740 | *mnaT* | 2.30 | N-acetyltransferase |
| ESA_RS08410 | N/A | 2.30 | hypothetical protein |
| ESA_RS11235 | *pflB* | 2.29 | formate acetyltransferase |
| ESA_RS05050 | *yohC* | 2.29 | YIP1 family protein |
| ESA_RS02405 | *iap* | 2.29 | Zn-dependent exopeptidase M28 |
| ESA_RS08370 | *uxaB* | 2.29 | altronate oxidoreductase |
| ESA_RS08065 | *ydfZ* | 2.27 | hypothetical protein |
| ESA_RS13855 | N/A | 2.27 | hypothetical protein |
| ESA_RS20735 | N/A | 2.27 | Unknown |
| ESA_RS09465 | *hycG* | 2.27 | formate hydrogenlyase |
| ESA_RS19525 | *uspB* | 2.26 | universal stress protein B |
| ESA_RS12540 | *ybdL* | 2.26 | pyridoxal phosphate-dependent aminotransferase |
| ESA_RS03590 | *fepD* | 2.26 | iron-enterobactin transporter membrane protein |
| ESA_RS06975 | *narJ* | 2.25 | nitrate reductase molybdenum cofactor assembly chaperone |
| ESA_RS11150 | *ycbJ* | 2.25 | hypothetical protein |
| ESA_RS13600 | *lpxP* | 2.24 | lipid A biosynthesis palmitoleoyl acyltransferase |
| ESA_RS08365 | N/A | 2.24 | N-acetyltransferase |
| ESA_RS02700 | *alaE* | 2.23 | L-alanine exporter AlaE |
| ESA_RS02475 | N/A | 2.22 | Unknown |
| ESA_RS09615 | *appA* | 2.21 | AppA family phytase/histidine-type acid phosphatase |
| ESA_RS05005 | *yohK* | 2.20 | CidB/LrgB family autolysis modulator |
| ESA_RS01240 | N/A | 2.20 | cystathionine beta-lyase |
| ESA_RS06970 | *narH* | 2.20 | nitrate reductase subunit beta |
| ESA_RS19880 | N/A | 2.20 | Unknown |
| ESA_RS14300 | *ompC* | 2.19 | porin OmpC |
| ESA_RS11930 | *aroG* | 2.18 | 3-deoxy-7-phosphoheptulonate synthase |
| ESA_RS08620 | *ssuC* | 2.18 | ABC transporter permease |
| ESA_RS03390 | N/A | 2.18 | peptidase M4 |
| ESA_RS11080 | *ssuB* | 2.16 | aliphatic sulfonates ABC transporter ATP-binding protein |
| ESA_RS02940 | *tyrA* | 2.16 | bifunctional chorismate mutase/prephenate dehydrogenase |
| ESA_RS05510 | *puuD* | 2.15 | gamma-glutamyl-gamma-aminobutyrate hydrolase |
| ESA_RS06945 | *ychO* | 2.13 | YchO family inverse autotransporter domain-containing protein |
| ESA_RS18005 | N/A | 2.13 | hypothetical protein |
| ESA_RS08890 | *yejA* | 2.12 | ABC transporter substrate binding protein |
| ESA_RS02430 | *ygbE* | 2.11 | membrane protein |
| ESA_RS03685 | *ulaR* | 2.11 | S-methyl-5-thioribose-1-phosphate isomerase |
| ESA_RS10240 | *ndh* | 2.11 | NADH dehydrogenase family protein |
| ESA_RS10900 | *yccJ* | 2.10 | hypothetical protein |
| ESA_RS14445 | *metQ* | 2.07 | methionine ABC transporter substrate-binding protein |
| ESA_RS13685 | *metP* | 2.07 | methionine import system permease |
| ESA_RS09395 | N/A | 2.07 | hypothetical protein |
| ESA_RS03210 | N/A | 2.07 | MerR family transcriptional regulator |
| ESA_RS11230 | *focA* | 2.06 | formate transporter FocA |
| ESA_RS08615 | N/A | 2.06 | sulfonate ABC transporter ATP-binding lipoprotein |
| ESA_RS08650 | *yohF* | 2.06 | short-chain dehydrogenase |
| ESA_RS18745 | *ynfE* | 2.05 | hypothetical protein |
| ESA_RS07990 | N/A | 2.05 | glycine/betaine ABC transporter substrate-binding protein |
| ESA_RS05680 | *vsr* | 2.05 | very short patch repair endonuclease |
| ESA_RS02690 | *ygaM* | 2.05 | membrane protein |
| ESA_RS09790 | *ydiZ* | 2.05 | hypothetical protein |
| ESA_RS15010 | *leuO* | 2.04 | transcriptional regulator |
| ESA_RS12740 | *purE* | 2.03 | 5-(carboxyamino)imidazole ribonucleotide mutase |
| ESA_RS18640 | *glnG* | 2.03 | nitrogen regulation protein NR(I) |
| ESA_RS04010 | *yfcZ* | 2.03 | hypothetical protein |
| ESA_RS15440 | N/A | 2.03 | ABC transporter ATP-binding protein |
| ESA_RS18020 | N/A | 2.02 | hypothetical protein |
| ESA_RS13810 | *pepT* | 2.02 | peptidase T |
| ESA_RS08110 | N/A | 2.01 | 5-methyltetrahydropteroyltriglutamate--homocysteine S-methyltransferase |
| ESA_RS21025 | N/A | 2.01 | Unknown |
| ESA_RS07980 | *proV* | 2.01 | glycine/betaine ABC transporter ATP-binding protein |
| ESA_RS06980 | *narI* | 2.00 | respiratory nitrate reductase subunit gamma |
| ESA_RS03600 | *fepB* | 2.00 | Fe2+-enterobactin ABC transporter substrate-binding protein |
| ESA_RS04120 | *cvpA* | 1.99 | colicin V production protein |
| ESA_RS20725 | N/A | 1.99 | Unknown |
| ESA_RS16670 | *yhcN* | 1.99 | DUF1471 domain-containing protein |
| ESA_RS20065 | *hofM* | 1.98 | DNA catabolic putative pilus assembly protein |
| ESA_RS21315 | N/A | 1.98 | Unknown |
| ESA_RS19185 | *malS* | 1.98 | alpha-amylase |
| ESA_RS05110 | *mdtA* | 1.95 | hypothetical protein |
| ESA_RS07895 | N/A | 1.95 | acetyltransferase |
| ESA_RS17190 | *metR* | 1.94 | HTH-type transcriptional regulator |
| ESA_RS19995 | *feoB* | 1.93 | ferrous iron transport protein B |
| ESA_RS16890 | *purD* | 1.92 | phosphoribosylamine--glycine ligase |
| ESA_RS10800 | *phoH* | 1.92 | phosphate starvation-inducible protein |
| ESA_RS10080 | *aphA1* | 1.92 | aminoglycoside phosphotransferase |
| ESA_RS09860 | *cho* | 1.91 | excinuclease |
| ESA_RS06180 | N/A | 1.88 | SCPU domain-containing protein |
| ESA_RS07525 | N/A | 1.88 | HK97 family phage prohead protease |
| ESA_RS10575 | *phoA* | 1.88 | phosphotransferase |
| ESA_RS09620 | *ybfF* | 1.87 | acyl-CoA esterase |
| ESA_RS05010 | N/A | 1.87 | hypothetical protein |
| ESA_RS18370 | N/A | 1.84 | LysR family transcriptional regulator |
| ESA_RS20230 | N/A | 1.83 | ribose-phosphate pyrophosphokinase |
| ESA_RS01250 | *exbD* | 1.83 | TonB system transport protein |
| ESA_RS12555 | N/A | 1.83 | acireductone dioxygenase |
| ESA_RS06570 | N/A | 1.83 | diguanylate phosphodiesterase |
| ESA_RS20070 | *hofN* | 1.83 | pilus assembly protein |
| ESA_RS04125 | N/A | 1.83 | amidophosphoribosyltransferase |
| ESA_RS18025 | *rhs* | 1.82 | type IV secretion protein |
| ESA_RS04230 | *ybiV* | 1.82 | sugar phosphatase |
| ESA_RS10230 | N/A | 1.82 | TetR/AcrR family transcriptional regulator |
| ESA_RS06960 | *narK* | 1.81 | NarK family nitrate/nitrite MFS transporter |
| ESA_RS18380 | N/A | 1.80 | NAD(P)H-dependent oxidoreductase |
| ESA_RS11145 | *elyC* | 1.80 | envelope biogenesis factor |
| ESA_RS14025 | N/A | 1.80 | phage head morphogenesis protein |
| ESA_RS02520 | N/A | 1.80 | N-acetyltransferase |
| ESA_RS20540 | N/A | 1.79 | Unknown |
| ESA_RS15785 | *gldA* | 1.79 | glycerol dehydrogenase |
| ESA_RS08025 | N/A | 1.79 | DUF1161 domain-containing protein |
| ESA_RS08155 | *urtE* | 1.78 | urea ABC transporter ATP-binding subunit |
| ESA_RS08320 | N/A | 1.78 | N-acetyltransferase |
| ESA_RS14760 | *gcd* | 1.77 | glucose/quinate/shikimate family membrane-bound PQQ-dependent dehydrogenase |
| ESA_RS20335 | N/A | 1.76 | prepilin peptidase |
| ESA_RS15420 | *kpsS* | 1.75 | capsular biosynthesis protein |
| ESA_RS01400 | N/A | 1.75 | DUF554 domain-containing protein |
| ESA_RS18105 | N/A | 1.74 | hypothetical protein |
| ESA_RS08630 | N/A | 1.73 | TonB-dependent receptor |
| ESA_RS12620 | *vgrG* | 1.73 | type VI secretion system tip protein |
| ESA_RS03205 | *glyA* | 1.73 | serine hydroxymethyltransferase |
| ESA_RS02950 | N/A | 1.73 | prephenate dehydratase |
| ESA_RS05810 | N/A | 1.73 | hypothetical protein |
| ESA_RS12745 | N/A | 1.72 | 5-(carboxyamino)imidazole ribonucleotide synthase |
| ESA_RS10100 |  | 1.71 | VOC family protein |
| ESA_RS10245 | N/A | 1.71 | hypothetical protein |
| ESA_RS13155 | N/A | 1.71 | hypothetical protein |
| ESA_RS10965 | N/A | 1.70 | CoA-binding protein |
| ESA_RS07985 | *osmW* | 1.70 | osmoprotectant ABC transporter permease |
| ESA_RS18120 | N/A | 1.70 | type VI secretion protein |
| ESA_RS06950 | *narL* | 1.70 | two-component system response regulator |
| ESA_RS07640 | N/A | 1.70 | DUF1311 domain-containing protein |
| ESA_RS11100 | *phoE* | 1.70 | phosphoporin |
| ESA_RS14270 | N/A | 1.69 | hypothetical protein |
| ESA_RS06035 | N/A | 1.69 | GNAT family N-acetyltransferase |
| ESA_RS09440 | N/A | 1.69 | formate dehydrogenase subunit alpha |
| ESA_RS15900 | N/A | 1.69 | HNH endonuclease |
| ESA_RS02470 | N/A | 1.69 | Unknown |
| ESA_RS20805 | N/A | 1.69 | Unknown |
| ESA_RS19890 | N/A | 1.68 | aspartate-semialdehyde dehydrogenase |
| ESA_RS02705 | N/A | 1.68 | hypothetical protein |
| ESA_RS13935 | N/A | 1.68 | peptidoglycan endopeptidase |
| ESA_RS15055 | *thiQ* | 1.68 | thiamine ABC transporter ATP-binding protein |
| ESA_RS18010 | N/A | 1.68 | hypothetical protein |
| ESA_RS01265 | N/A | 1.68 | hypothetical protein |
| ESA_RS16155 | N/A | 1.68 | DUF1120 domain-containing protein |
| ESA_RS10805 | *efeB* | 1.67 | deferrochelatase/peroxidase |
| ESA_RS06450 | N/A | 1.67 | hypothetical protein |
| ESA_RS05975 | N/A | 1.67 | cystine ABC transporter substrate-binding protein |
| ESA_RS14055 | N/A | 1.67 | hypothetical protein |
| ESA_RS07740 | N/A | 1.67 | 2-hydroxyacid dehydrogenase |
| ESA_RS18040 | N/A | 1.66 | DUF2169 domain-containing protein |
| ESA_RS05560 | N/A | 1.66 | FMN/FAD transporter |
| ESA_RS04865 | N/A | 1.66 | phage resistance protein |
| ESA_RS20815 | N/A | 1.65 | Unknown |
| ESA_RS03430 | N/A | 1.65 | phosphoribosylglycinamide formyltransferase |
| ESA_RS18045 | *vgrG* | 1.65 | type VI secretion system tip protein |
| ESA_RS09655 | N/A | 1.65 | 3-deoxy-7-phosphoheptulonate synthase |
| ESA_RS20330 | N/A | 1.65 | bacterioferritin |
| ESA_RS19875 | *fic* | 1.64 | cell filamentation protein |
| ESA_RS18070 | *tssG* | 1.64 | type VI secretion system baseplate subunit |
| ESA_RS10885 | N/A | 1.64 | pyrimidine utilization transport protein G |
| ESA_RS19700 | N/A | 1.64 | TRAP transporter large permease |
| ESA_RS04235 | N/A | 1.64 | SLC13 family permease |
| ESA_RS12750 | N/A | 1.63 | PLP-dependent transferase |
| ESA_RS20235 | N/A | 1.63 | nicotinate phosphoribosyltransferase |
| ESA_RS00475 | N/A | 1.63 | hypothetical protein |
| ESA_RS20955 | N/A | 1.63 | Unknown |
| ESA_RS16095 | *sstT* | 1.63 | serine/threonine transporter |
| ESA_RS20970 | N/A | 1.63 | Unknown |
| ESA_RS11540 | *gsiA* | 1.62 | glutathione ABC transporter ATP-binding protein |
| ESA_RS20975 | N/A | 1.62 | Unknown |
| ESA_RS11220 | N/A | 1.62 | DUF421 domain-containing protein |
| ESA_RS10470 | N/A | 1.62 | MFS transporter |
| ESA_RS11900 | *modF* | 1.62 | molybdate ABC transporter ATP-binding protein |
| ESA_RS06955 | *narX* | 1.60 | nitrate/nitrite two-component system sensor histidine kinase |
| ESA_RS09405 | N/A | 1.60 | hypothetical protein |
| ESA_RS20695 | N/A | 1.60 | Unknown |
| ESA_RS10355 | N/A | 1.59 | DUF3053 domain-containing protein |
| ESA_RS12545 | N/A | 1.59 | methylthioribulose 1-phosphate dehydratase |
| ESA_RS10695 | N/A | 1.59 | EAL domain-containing protein |
| ESA_RS05580 | N/A | 1.59 | DUF3396 domain-containing protein |
| ESA_RS10165 | N/A | 1.59 | peptidase T |
| ESA_RS21105 | N/A | 1.58 | Unknown |
| ESA_RS21375 | N/A | 1.58 | DUF551 domain-containing protein |
| ESA_RS21150 | N/A | 1.58 | Unknown |
| ESA_RS20860 | N/A | 1.58 | Unknown |
| ESA_RS18050 | N/A | 1.58 | accessory protein |
| ESA_RS14170 | N/A | 1.58 | hypothetical protein |
| ESA_RS06445 | N/A | 1.57 | Unknown |
| ESA_RS07585 | N/A | 1.57 | glycoside hydrolase family 65 protein |
| ESA_RS07995 | *osmY* | 1.57 | osmoprotectant ABC transporter permease |
| ESA_RS18035 | N/A | 1.56 | DUF4150 domain-containing protein |
| ESA_RS21030 | N/A | 1.56 | Unknown |
| ESA_RS08690 | *arcB* | 1.55 | ornithine cyclodeaminase |
| ESA_RS14845 | N/A | 1.55 | alpha-N-arabinofuranosidase |
| ESA_RS06965 | N/A | 1.54 | nitrate reductase subunit alpha |
| ESA_RS09845 | *bamE* | 1.54 | outer membrane protein assembly factor |
| ESA_RS03900 | N/A | 1.54 | cupin domain-containing protein |
| ESA_RS18065 | *tssH* | 1.54 | type VI secretion system ATPase |
| ESA_RS01255 | N/A | 1.54 | NAD(P)-dependent oxidoreductase |
| ESA_RS13425 | N/A | 1.54 | shikimate kinase |
| ESA_RS02465 | *nlpD* | 1.54 | murein hydrolase activator |
| ESA_RS21165 | N/A | 1.54 | Unknown |
| ESA_RS07270 | *fsaA* | 1.53 | fructose-6-phosphate aldolase |
| ESA_RS05675 | N/A | 1.53 | DNA cytosine methylase |
| ESA_RS03945 | N/A | 1.53 | membrane protein |
| ESA_RS11390 | *artQ* | 1.53 | arginine transporter permease subunit |
| ESA_RS04250 | *lrhA* | 1.53 | transcriptional regulator |
| ESA_RS06715 | N/A | 1.52 | SpoVR family protein |
| ESA_RS00600 | N/A | 1.51 | hypothetical protein |
| ESA_RS19915 | N/A | 1.51 | glycogen phosphorylase |
| ESA_RS09015 | N/A | 1.51 | type 1 fimbrial protein |
| ESA_RS18110 | N/A | 1.50 | hypothetical protein |
| ESA_RS10475 | N/A | 1.50 | glutaredoxin 2 |
| ESA_RS05825 | N/A | 1.50 | hypothetical protein |
| ESA_RS10895 | N/A | 1.50 | NAD(P)H:quinone oxidoreductase |
| ESA_RS08975 | N/A | 1.49 | 3-oxoacyl-ACP reductase |
| ESA_RS17290 | N/A | 1.49 | class I adenylate cyclase |
| ESA_RS02815 | N/A | 1.49 | hypothetical protein |
| ESA_RS02735 | N/A | 1.49 | DUF1493 domain-containing protein |
| ESA_RS11210 | N/A | 1.48 | 3-phosphoshikimate 1-carboxyvinyltransferase |
| ESA_RS11395 | *artM* | 1.48 | arginine transporter permease subunit |
| ESA_RS00590 | N/A | 1.48 | hypothetical protein |
| ESA_RS09590 | *iscS* | 1.48 | cysteine desulfurase |
| ESA_RS16885 | N/A | 1.48 | bifunctional phosphoribosylaminoimidazolecarboxamide formyltransferase |
| ESA_RS19380 | *dppF* | 1.46 | dipeptide ABC transporter ATP-binding protein |
| ESA_RS06270 | *cutC* | 1.46 | copper homeostasis protein |
| ESA_RS01960 | N/A | 1.46 | hemolysin III family protein |
| ESA_RS00260 | N/A | 1.46 | methionine synthase |
| ESA_RS11240 | *pflA* | 1.45 | pyruvate formate lyase 1-activating protein |
| ESA_RS09525 | *flhA* | 1.45 | formate hydrogenlyase transcriptional activator |
| ESA_RS17990 | *rhs* | 1.45 | type IV secretion protein |
| ESA_RS03650 | N/A | 1.45 | MFS transporter |
| ESA_RS20055 | *nudE* | 1.45 | ADP compounds hydrolase |
| ESA_RS18115 | N/A | 1.45 | hypothetical protein |
| ESA_RS02025 | N/A | 1.45 | hypothetical protein |
| ESA_RS07280 | N/A | 1.45 | enoyl-CoA hydratase |
| ESA_RS04225 | N/A | 1.45 | YfbU family protein |
| ESA_RS11535 | *gsiB* | 1.44 | glutathione ABC transporter substrate-binding protein |
| ESA_RS17660 | *rraA* | 1.44 | ribonuclease E activity regulator |
| ESA_RS19845 | N/A | 1.44 | N-acetyltransferase |
| ESA_RS04345 | N/A | 1.43 | 2-succinyl-6-hydroxy-2,4-cyclohexadiene-1-carboxylate synthase |
| ESA_RS11385 | N/A | 1.43 | arginine ABC transporter substrate-binding protein |
| ESA_RS15600 | N/A | 1.43 | membrane protein |
| ESA_RS07865 | N/A | 1.43 | alkene reductase |
| ESA_RS01575 | N/A | 1.43 | hypothetical protein |
| ESA_RS08170 | N/A | 1.43 | IS3 family transposase |
| ESA_RS03215 | N/A | 1.43 | 4-carboxymuconolactone decarboxylase |
| ESA_RS01260 | N/A | 1.43 | HTH domain-containing protein |
| ESA_RS15575 | *fhuF* | 1.43 | hydroxamate siderophore iron reductase |
| ESA_RS10050 | N/A | 1.42 | hypothetical protein |
| ESA_RS05820 | N/A | 1.42 | hypothetical protein |
| ESA_RS10810 | *efeO* | 1.42 | iron uptake system protein |
| ESA_RS01245 | *exbB* | 1.42 | biopolymer transporter |
| ESA_RS04960 | N/A | 1.42 | MFS transporter |
| ESA_RS02080 | *ygeA* | 1.42 | aspartate/glutamate racemase |
| ESA_RS11945 | *pnuC* | 1.42 | nicotinamide riboside transporter |
| ESA_RS08720 | N/A | 1.42 | NAD(P)-dependent oxidoreductase |
| ESA_RS11215 | N/A | 1.42 | 3-phosphoserine/phosphohydroxythreonine aminotransferase |
| ESA_RS20000 | *feoA* | 1.41 | iron transporter |
| ESA_RS08645 | N/A | 1.41 | glycosidase |
| ESA_RS18755 | N/A | 1.41 | hypothetical protein |
| ESA_RS07420 | N/A | 1.41 | replication protein |
| ESA_RS12835 | N/A | 1.41 | Kef family K(+) transporter |
| ESA_RS10855 | N/A | 1.41 | pyrimidine utilization protein A |
| ESA_RS08390 | N/A | 1.41 | hybrid sensor histidine kinase/response regulator |
| ESA_RS02695 | N/A | 1.40 | hypothetical protein |
| ESA_RS13780 | *pqqB* | 1.40 | pyrroloquinoline quinone biosynthesis protein |
| ESA_RS18645 | *ntrB* | 1.40 | nitrogen regulation protein NR(II) |
| ESA_RS11515 | N/A | 1.40 | PQQ-dependent sugar dehydrogenase |
| ESA_RS00235 | N/A | 1.40 | homoserine O-succinyltransferase |
| ESA_RS01895 | N/A | 1.40 | 5-formyltetrahydrofolate cyclo-ligase |
| ESA_RS08540 | N/A | 1.39 | hypothetical protein |
| ESA_RS06935 | N/A | 1.39 | nitrate reductase |
| ESA_RS07890 | N/A | 1.39 | 50S ribosomal protein L7/L12-serine acetyltransferase |
| ESA_RS00285 | N/A | 1.38 | hypothetical protein |
| ESA_RS13925 | N/A | 1.38 | hypothetical protein |
| ESA_RS15270 | N/A | 1.38 | hypothetical protein |
| ESA_RS08545 | N/A | 1.38 | NAD(P)-dependent oxidoreductase |
| ESA_RS01070 | *yjfF* | 1.38 | sugar ABC transporter permease |
| ESA_RS15605 | N/A | 1.38 | hypothetical protein |
| ESA_RS00595 | *crfC* | 1.37 | clamp-binding protein |
| ESA_RS07515 | N/A | 1.37 | terminase large subunit |
| ESA_RS11855 | N/A | 1.37 | kinase inhibitor |
| ESA_RS18055 | N/A | 1.37 | hypothetical protein |
| ESA_RS08730 |  | 1.36 | ABC transporter substrate-binding protein |
| ESA_RS10040 | N/A | 1.36 | DUF4303 domain-containing protein |
| ESA_RS03305 | N/A | 1.35 | hypothetical protein |
| ESA_RS10070 | N/A | 1.35 | hypothetical protein |
| ESA_RS08405 | N/A | 1.35 | DUF3131 domain-containing protein |
| ESA_RS19295 | N/A | 1.35 | LacI family DNA-binding transcriptional regulator |
| ESA_RS04330 | N/A | 1.34 | DUF883 domain-containing protein |
| ESA_RS03855 | N/A | 1.34 | divalent metal cation transporter |
| ESA_RS09520 | *hypE* | 1.34 | hydrogenase expression/formation protein |
| ESA_RS18160 | *TagF* | 1.34 | type VI secretion system-associated protein |
| ESA_RS06065 | N/A | 1.33 | MFS transporter |
| ESA_RS06175 | N/A | 1.33 | fimbrial biogenesis outer membrane usher protein |
| ESA_RS10130 | N/A | 1.33 | methyl-accepting chemotaxis protein |
| ESA_RS17210 | *rhtB* | 1.33 | homoserine/homoserine lactone efflux protein |
| ESA_RS08940 | *uspE* | 1.33 | universal stress protein |
| ESA_RS08010 | N/A | 1.33 | dimethyl sulfoxide reductase subunit H |
| ESA_RS01405 | *speC* | 1.33 | ornithine decarboxylase |
| ESA_RS09665 | *cdgR* | 1.32 | cyclic di-GMP regulator |
| ESA_RS11530 | *gsiC* | 1.32 | glutathione ABC transporter permease |
| ESA_RS09805 | N/A | 1.32 | 2-deoxyglucose-6-phosphatase |
| ESA_RS12580 | *entD* | 1.32 | enterobactin synthase subunit |
| ESA_RS03745 | *cysM* | 1.32 | cysteine synthase |
| ESA_RS20730 | N/A | 1.32 | Unknown |
| ESA_RS06990 | N/A | 1.31 | oxidoreductase |
| ESA_RS04785 | N/A | 1.31 | hypothetical protein |
| ESA_RS16340 | *nlpI* | 1.31 | lipoprotein |
| ESA_RS09340 | *tssG* | 1.31 | type VI secretion system baseplate subunit |
| ESA_RS06280 | N/A | 1.31 | hypothetical protein |
| ESA_RS14005 | N/A | 1.30 | hypothetical protein |
| ESA_RS07960 | N/A | 1.30 | MFS transporter |
| ESA_RS08785 | N/A | 1.30 | dimethyl sulfoxide reductase subunit A |
| ESA_RS13800 | *pqqF* | 1.30 | pyrroloquinoline quinone biosynthesis protein |
| ESA_RS13670 | N/A | 1.30 | phenolic acid decarboxylase |
| ESA_RS04535 | N/A | 1.29 | recombinase |
| ESA_RS10770 | *stdB* | 1.29 | fimbrial biogenesis outer membrane usher protein |
| ESA_RS19905 | *glgC* | 1.29 | glucose-1-phosphate adenylyltransferase |
| ESA_RS15260 | N/A | 1.29 | chitinase |
| ESA_RS09865 | *ves* | 1.29 | protein |
| ESA_RS14830 | *pdhR* | 1.28 | pyruvate dehydrogenase complex transcriptional repressor |
| ESA_RS19630 | *pcoC* | 1.28 | copper resistance system chaperone |
| ESA_RS07825 | N/A | 1.28 | alpha/beta hydrolase |
| ESA_RS03195 | *glnB* | 1.28 | nitrogen regulatory protein P-II |
| ESA_RS17975 | N/A | 1.28 | hypothetical protein |
| ESA_RS20720 | N/A | 1.28 | Unknown |
| ESA_RS13675 | N/A | 1.28 | glycoside hydrolase family 15 |
| ESA_RS08430 | N/A | 1.28 | hypothetical protein |
| ESA_RS08600 | N/A | 1.27 | DUF1176 domain-containing protein |
| ESA_RS09035 | N/A | 1.27 | fimbrial biogenesis outer membrane usher protein |
| ESA_RS05805 | N/A | 1.27 | hypothetical protein |
| ESA_RS03435 | N/A | 1.27 | phosphoribosylformylglycinamidine cyclo-ligase |
| ESA_RS13795 | *pqqE* | 1.27 | pyrroloquinoline quinone biosynthesis protein |
| ESA_RS13515 | N/A | 1.27 | autotransporter outer membrane beta-barrel domain-containing protein |
| ESA_RS18095 | N/A | 1.26 | serine/threonine-protein phosphatase |
| ESA_RS10350 | *maf* | 1.26 | septum formation inhibitor |
| ESA_RS07815 | N/A | 1.26 | YdcF family protein |
| ESA_RS11665 | N/A | 1.26 | DUF1471 domain-containing protein |
| ESA_RS15610 | N/A | 1.26 | MarR family transcriptional regulator |
| ESA_RS09970 | N/A | 1.26 | PrkA family serine protein kinase |
| ESA_RS20655 | N/A | 1.26 | Unknown |
| ESA_RS17460 | N/A | 1.25 | ATP-dependent protease |
| ESA_RS00180 | N/A | 1.25 | DUF494 domain-containing protein |
| ESA_RS05865 | N/A | 1.25 | class I SAM-dependent methyltransferase |
| ESA_RS15985 | N/A | 1.25 | YhcH/YjgK/YiaL family protein |
| ESA_RS15895 | N/A | 1.25 | YhcH/YjgK/YiaL family protein |
| ESA_RS01370 | N/A | 1.25 | DUF2138 domain-containing protein |
| ESA_RS19895 | N/A | 1.25 | 1,4-alpha-glucan branching enzyme |
| ESA_RS17610 | *metL* | 1.24 | bifunctional aspartate kinase/homoserine dehydrogenase II |
| ESA_RS20675 | N/A | 1.24 | Unknown |
| ESA_RS12890 | N/A | 1.24 | fimbrial biogenesis outer membrane usher protein |
| ESA_RS20770 | N/A | 1.24 | Unknown |
| ESA_RS08560 | N/A | 1.23 | AraC family transcriptional regulator |
| ESA_RS12475 | N/A | 1.23 | alpha-glucosidase |
| ESA_RS04605 | N/A | 1.23 | Unknown |
| ESA_RS19375 | *dppD* | 1.22 | dipeptide ABC transporter ATP-binding protein |
| ESA_RS04645 | N/A | 1.21 | type II toxin-antitoxin system RelE/ParE family toxin |
| ESA_RS18140 | N/A | 1.21 | Unknown |
| ESA_RS08610 | N/A | 1.21 | TauD/TfdA family dioxygenase |
| ESA_RS10570 | N/A | 1.21 | molecular chaperone |
| ESA_RS02930 | N/A | 1.21 | PepSY domain-containing protein |
| ESA_RS11300 | *clpA* | 1.21 | ATP-dependent Clp protease ATP-binding subunit |
| ESA_RS19910 | *glgA* | 1.21 | glycogen synthase |
| ESA_RS20865 | N/A | 1.21 | Unknown |
| ESA_RS07735 | N/A | 1.21 | META domain-containing protein |
| ESA_RS08910 | N/A | 1.21 | putative FMN-dependent luciferase-like monooxygenase |
| ESA_RS15390 | N/A | 1.21 | Trp operon repressor |
| ESA_RS14385 | N/A | 1.21 | SAM-dependent methyltransferase |
| ESA_RS09850 | *nadE* | 1.20 | NAD(+) synthetase |
| ESA_RS18075 | *tssF* | 1.20 | type VI secretion system baseplate subunit |
| ESA_RS07690 | N/A | 1.20 | hypothetical protein |
| ESA_RS17565 | N/A | 1.20 | acetylornithine deacetylase |
| ESA_RS11350 | N/A | 1.20 | ubiquinone-dependent pyruvate dehydrogenase |
| ESA_RS11245 | N/A | 1.20 | MFS transporter |
| ESA_RS11525 | *gsiD* | 1.20 | glutathione ABC transporter permease |
| ESA_RS14370 | N/A | 1.20 | class I SAM-dependent methyltransferase |
| ESA_RS13455 | N/A | 1.19 | sugar porter family MFS transporter |
| ESA_RS04855 | *yejB* | 1.19 | microcin C ABC transporter permease |
| ESA_RS09005 | *manA* | 1.19 | mannose-6-phosphate isomerase |
| ESA_RS20710 | N/A | 1.19 | Unknown |
| ESA_RS05995 | *sdiA* | 1.19 | transcriptional regulator |
| ESA_RS01585 | N/A | 1.19 | mismatch-specific DNA-glycosylase |
| ESA_RS20830 | N/A | 1.18 | Unknown |
| ESA_RS08885 | N/A | 1.18 | glycoporin |
| ESA_RS20885 | N/A | 1.17 | Unknown |
| ESA_RS17995 | N/A | 1.17 | DUF1795 domain-containing protein |
| ESA_RS05255 | N/A | 1.17 | molecular chaperone |
| ESA_RS02095 | N/A | 1.16 | LacI family DNA-binding transcriptional regulator |
| ESA_RS13690 | N/A | 1.16 | methionine ABC transporter ATP-binding protein |
| ESA_RS05665 | *ompC* | 1.16 | porin |
| ESA_RS17850 | N/A | 1.15 | hypothetical protein |
| ESA_RS09570 | *pykF* | 1.15 | pyruvate kinase |
| ESA_RS01105 | N/A | 1.15 | cytochrome b562 |
| ESA_RS18185 | N/A | 1.15 | hypothetical protein |
| ESA_RS04850 | N/A | 1.15 | microcin C ABC transporter permease |
| ESA_RS05570 | N/A | 1.14 | VRR-NUC domain-containing protein |
| ESA_RS16690 | *aaeB* | 1.14 | p-hydroxybenzoic acid efflux pump subunit |
| ESA_RS02925 | N/A | 1.14 | DUF2946 domain-containing protein |
| ESA_RS01625 | N/A | 1.14 | undecaprenyl-diphosphatase |
| ESA_RS00270 | N/A | 1.14 | hypothetical protein |
| ESA_RS08685 | N/A | 1.13 | peptidase M20 |
| ESA_RS05575 | N/A | 1.13 | DUF3396 domain-containing protein |
| ESA_RS07225 | N/A | 1.13 | phosphatidylglycerophosphatase B |
| ESA_RS07055 | *oppA* | 1.13 | oligopeptide ABC transporter substrate-binding protein |
| ESA_RS09625 | N/A | 1.13 | FAD-binding oxidoreductase |
| ESA_RS20500 | N/A | 1.13 | Unknown |
| ESA_RS04545 | N/A | 1.13 | 3'-5' exoribonuclease |
| ESA_RS14380 | N/A | 1.12 | murein transglycosylase D |
| ESA_RS05235 | *mdtC* | 1.12 | multidrug resistance protein |
| ESA_RS08510 | N/A | 1.12 | UV damage repair endonuclease |
| ESA_RS00980 | N/A | 1.12 | DUF1311 domain-containing protein |
| ESA_RS21075 | N/A | 1.12 | Unknown |
| ESA_RS05035 | N/A | 1.12 | MBL fold metallo-hydrolase |
| ESA_RS05840 | N/A | 1.11 | XRE family transcriptional regulator |
| ESA_RS16335 | N/A | 1.11 | DEAD/DEAH family ATP-dependent RNA helicase |
| ESA_RS11770 | N/A | 1.11 | lysylphosphatidylglycerol synthetase family protein |
| ESA_RS16130 | *mzrA* | 1.11 | modulator protein |
| ESA_RS07025 | N/A | 1.10 | UDP-glucose/GDP-mannose dehydrogenase family protein |
| ESA_RS04160 | N/A | 1.10 | RES domain-containing protein |
| ESA_RS02935 | N/A | 1.10 | 3-deoxy-7-phosphoheptulonate synthase |
| ESA_RS18375 | N/A | 1.10 | hypothetical protein |
| ESA_RS19540 | N/A | 1.10 | transposase |
| ESA_RS11510 | N/A | 1.10 | glutathione S-transferase family protein |
| ESA_RS20825 | N/A | 1.09 | Unknown |
| ESA_RS14770 | N/A | 1.09 | hypothetical protein |
| ESA_RS11775 | N/A | 1.09 | BAX inhibitor (BI)-1/YccA family protein |
| ESA_RS15510 | N/A | 1.09 | metal-dependent hydrolase |
| ESA_RS09580 | N/A | 1.09 | LysM peptidoglycan-binding domain-containing protein |
| ESA_RS16285 | N/A | 1.09 | hypothetical protein |
| ESA_RS12755 | N/A | 1.09 | CBS domain-containing protein |
| ESA_RS09555 | N/A | 1.09 | FAD-binding oxidoreductase |
| ESA_RS01790 | N/A | 1.08 | YqgE/AlgH family protein |
| ESA_RS20555 | N/A | 1.08 | Unknown |
| ESA_RS09595 | *sppA* | 1.08 | signal peptide peptidase |
| ESA_RS19585 | N/A | 1.08 | efflux RND transporter periplasmic adaptor subunit |
| ESA_RS15865 | N/A | 1.08 | DUF898 domain-containing protein |
| ESA_RS15955 | N/A | 1.08 | DUF898 domain-containing protein |
| ESA_RS10610 | N/A | 1.08 | DUF1983 domain-containing protein |
| ESA_RS10765 | N/A | 1.08 | fimbrial protein |
| ESA_RS04615 | N/A | 1.08 | phage N-6-adenine-methyltransferase |
| ESA_RS00525 | N/A | 1.07 | NCS2 family permease |
| ESA_RS09975 | N/A | 1.07 | hypothetical protein |
| ESA_RS03020 | N/A | 1.07 | thiamine pyrophosphate-requiring protein |
| ESA_RS02945 | N/A | 1.07 | SMP-30/gluconolactonase/LRE family protein |
| ESA_RS05505 | N/A | 1.07 | NAD(P)-dependent oxidoreductase |
| ESA_RS11005 | N/A | 1.07 | macrodomain Ter protein |
| ESA_RS20495 | N/A | 1.07 | Unknown |
| ESA_RS10065 | N/A | 1.07 | hypothetical protein |
| ESA_RS12630 | N/A | 1.07 | phage head morphogenesis protein |
| ESA_RS01785 | *ruvX* | 1.07 | Holliday junction resolvase |
| ESA_RS12675 | N/A | 1.06 | antitermination protein |
| ESA_RS21290 | N/A | 1.06 | Unknown |
| ESA_RS03505 | N/A | 1.06 | esterase |
| ESA_RS00335 | N/A | 1.06 | N-acetyltransferase |
| ESA_RS12825 | N/A | 1.06 | Cys-tRNA(Pro) deacylase |
| ESA_RS21070 | N/A | 1.06 | Unknown |
| ESA_RS09350 | N/A | 1.06 | hypothetical protein |
| ESA_RS03395 | N/A | 1.06 | exodeoxyribonuclease 7 large subunit |
| ESA_RS18085 | N/A | 1.06 | hypothetical protein |
| ESA_RS18165 | *tssM* | 1.06 | type VI secretion system membrane subunit |
| ESA_RS14840 | N/A | 1.06 | MFS transporter |
| ESA_RS12550 | N/A | 1.06 | acireductone synthase |
| ESA_RS08380 | *treF* | 1.06 | alpha,alpha-trehalase |
| ESA_RS20910 | N/A | 1.05 | Unknown |
| ESA_RS00205 | N/A | 1.05 | gamma carbonic anhydrase family protein |
| ESA_RS17205 | N/A | 1.05 | lysophospholipase |
| ESA_RS12925 | N/A | 1.05 | primosomal protein |
| ESA_RS13930 | N/A | 1.05 | phage tail protein |
| ESA_RS15570 | N/A | 1.05 | GGDEF domain-containing protein |
| ESA_RS19335 | N/A | 1.04 | organic hydroperoxide resistance protein |
| ESA_RS14175 | N/A | 1.04 | hypothetical protein |
| ESA_RS19065 | N/A | 1.04 | hypothetical protein |
| ESA_RS04500 | N/A | 1.04 | hypothetical protein |
| ESA_RS21145 | N/A | 1.04 | Unknown |
| ESA_RS13730 | N/A | 1.04 | hypothetical protein |
| ESA_RS10495 | *bssS* | 1.04 | biofilm formation regulatory protein |
| ESA_RS05550 | N/A | 1.04 | hypothetical protein |
| ESA_RS17985 | N/A | 1.04 | hypothetical protein |
| ESA_RS19900 | N/A | 1.03 | glycogen debranching enzyme |
| ESA_RS13280 | N/A | 1.03 | aldo/keto reductase |
| ESA_RS09785 | N/A | 1.03 | 6-phosphofructokinase II |
| ESA_RS09585 | *sufE* | 1.03 | cysteine desulfuration protein |
| ESA_RS17515 | *btuB* | 1.03 | vitamin B12 transporter |
| ESA_RS11090 | N/A | 1.03 | nicotinate phosphoribosyltransferase |
| ESA_RS18155 | *tssA* | 1.03 | type VI secretion system protein |
| ESA_RS05230 | N/A | 1.03 | MFS transporter |
| ESA_RS18145 | N/A | 1.03 | type VI secretion system contractile sheath large subunit |
| ESA_RS05705 | N/A | 1.03 | mannosyl-3-phosphoglycerate phosphatase-related protein |
| ESA_RS05595 | N/A | 1.02 | diguanylate phosphodiesterase |
| ESA_RS11340 | N/A | 1.02 | hydroxylamine reductase |
| ESA_RS18080 | *tssE* | 1.02 | type VI secretion system baseplate subunit |
| ESA_RS03235 | N/A | 1.02 | nickel transporter |
| ESA_RS00410 | *zur* | 1.02 | transcriptional regulator |
| ESA_RS19680 | N/A | 1.02 | VUT family protein |
| ESA_RS11695 | N/A | 1.02 | type 1 fimbrial protein |
| ESA_RS08675 | N/A | 1.02 | ABC transporter permease |
| ESA_RS12505 | N/A | 1.01 | GntP family permease |
| ESA_RS20835 | N/A | 1.01 | Unknown |
| ESA_RS04690 | N/A | 1.01 | phage portal protein |
| ESA_RS05260 | N/A | 1.01 | DNA-3-methyladenine glycosylase 2 |
| ESA_RS20535 | N/A | 1.01 | Unknown |
| ESA_RS01940 | N/A | 1.01 | NAD(P)-dependent oxidoreductase |
| ESA_RS08385 | N/A | 1.00 | glycoside hydrolase family 2 |
| ESA_RS09420 | N/A | 1.00 | formate dehydrogenase subunit alpha |
| ESA_RS01420 | N/A | 1.00 | hypothetical protein |
| ESA_RS06655 | N/A | -1.00 | long-chain acyl-CoA synthetase |
| ESA_RS03460 | N/A | -1.00 | putative metalloprotease |
| ESA_RS13260 | *thiI* | -1.00 | thiamine biosynthesis protein |
| ESA_RS00895 | N/A | -1.01 | methyl-accepting chemotaxis protein II, aspartate sensor receptor |
| ESA_RS06505 | N/A | -1.01 | IclR family transcriptional regulator, KDG regulon repressor |
| ESA_RS02435 | *ftsB* | -1.01 | cell division protein |
| ESA_RS00345 | *malG* | -1.01 | maltose/maltodextrin transport system permease protein |
| ESA_RS01825 | *speA* | -1.02 | biosynthetic arginine decarboxylase |
| ESA_RS19720 | N/A | -1.02 | putative membrane protein |
| ESA_RS01095 | N/A | -1.02 | ribosome-associated protein |
| ESA_RS11010 | N/A | -1.02 | Lon-like ATP-dependent protease |
| ESA_RS04255 | N/A | -1.02 | NADH-quinone oxidoreductase subunit A |
| ESA_RS04420 | N/A | -1.03 | ribonucleoside-diphosphate reductase beta chain |
| ESA_RS06070 | N/A | -1.03 | Unknown |
| ESA_RS03110 | *lepA* | -1.03 | GTP-binding protein |
| ESA_RS09230 | N/A | -1.03 | murein DD-endopeptidase |
| ESA_RS05520 | N/A | -1.04 | putrescine importer |
| ESA_RS09935 | *ansA* | -1.04 | L-asparaginase |
| ESA_RS13290 | N/A | -1.04 | thiamine-monophosphate kinase |
| ESA_RS19180 | *avtA* | -1.04 | valine--pyruvate aminotransferase |
| ESA_RS04270 | N/A | -1.04 | NADH-quinone oxidoreductase subunit E |
| ESA_RS18535 | N/A | -1.05 | ribose transport system ATP-binding protein |
| ESA_RS12730 | N/A | -1.05 | peptidyl-prolyl cis-trans isomerase B (cyclophilin B) |
| ESA_RS06540 | N/A | -1.05 | Unknown |
| ESA_RS05540 | N/A | -1.05 | DNA gyrase inhibitor |
| ESA_RS05270 | N/A | -1.06 | uridine kinase |
| ESA_RS00955 | N/A | -1.06 | regulator of cell morphogenesis and NO signaling |
| ESA_RS16995 | *rplJ* | -1.06 | large subunit ribosomal protein L10 |
| ESA_RS01660 | *ribB* | -1.06 | 3,4-dihydroxy 2-butanone 4-phosphate synthase |
| ESA_RS01710 | N/A | -1.06 | topoisomerase IV subunit B |
| ESA_RS03060 | N/A | -1.07 | RNA methyltransferase, TrmH family |
| ESA_RS13115 | *cysM* | -1.07 | cysteine synthase A |
| ESA_RS11755 | N/A | -1.07 | Unknown |
| ESA_RS12840 | N/A | -1.07 | inosine kinase |
| ESA_RS12110 | N/A | -1.07 | Unknown |
| ESA_RS02965 | *bamD* | -1.08 | outer membrane protein assembly factor |
| ESA_RS15215 | N/A | -1.08 | non-specific riboncleoside hydrolase |
| ESA_RS13620 | N/A | -1.08 | c-di-GMP phosphodiesterase |
| ESA_RS06335 | *ruvA* | -1.08 | holliday junction DNA helicase |
| ESA_RS16990 | *rplL* | -1.08 | large subunit ribosomal protein L7/L12 |
| ESA_RS19750 | *ydcR* | -1.08 | GntR family transcriptional regulator / MocR family aminotransferase |
| ESA_RS19205 | N/A | -1.09 | D-xylose transport system ATP-binding protein |
| ESA_RS19725 | *rsmD* | -1.09 | 16S rRNA (guanine966-N2)-methyltransferase |
| ESA_RS00135 | *rplQ* | -1.09 | large subunit ribosomal protein L17 |
| ESA_RS12415 | N/A | -1.09 | uncharacterized protein |
| ESA_RS13900 | N/A | -1.09 | Unknown |
| ESA_RS17245 | N/A | -1.09 | Unknown |
| ESA_RS08340 | *glsB* | -1.09 | glutaminase |
| ESA_RS19145 | N/A | -1.10 | pyridoxine 4-dehydrogenase |
| ESA_RS08705 | N/A | -1.10 | Unknown |
| ESA_RS20310 | N/A | -1.10 | small subunit ribosomal protein S7 |
| ESA_RS12850 | *garK* | -1.10 | glycerate kinase |
| ESA_RS18480 | N/A | -1.10 | F-type H+-transporting ATPase subunit a |
| ESA_RS01685 | N/A | -1.11 | outer membrane protein |
| ESA_RS07685 | *ttcA* | -1.11 | tRNA 2-thiocytidine biosynthesis protein |
| ESA_RS13220 | N/A | -1.11 | cytochrome o ubiquinol oxidase subunit I |
| ESA_RS11650 | N/A | -1.12 | 23S rRNA (adenine1618-N6)-methyltransferase |
| ESA_RS15150 | *kefF* | -1.12 | glutathione-regulated potassium-efflux system ancillary protein |
| ESA_RS01080 | N/A | -1.12 | fructose-1,6-bisphosphatase I |
| ESA_RS13915 | N/A | -1.12 | polyisoprenyl-phosphate glycosyltransferase |
| ESA_RS20175 | N/A | -1.13 | peptidyl-prolyl cis-trans isomerase A (cyclophilin A) |
| ESA_RS15090 | *hepA* | -1.13 | ATP-dependent helicase |
| ESA_RS12055 | N/A | -1.13 | 2-oxoglutarate dehydrogenase E2 component (dihydrolipoamide succinyltransferase) |
| ESA_RS02170 | N/A | -1.13 | phosphatidylglycerol:prolipoprotein diacylglycerol transferase |
| ESA_RS03895 | *alaC* | -1.14 | alanine transaminase |
| ESA_RS04870 | *spr* | -1.14 | lipoprotein |
| ESA_RS00945 | *fklB* | -1.15 | FKBP-type peptidyl-prolyl cis-trans isomerase |
| ESA_RS00385 | *plsB* | -1.15 | glycerol-3-phosphate 1-O-acyltransferase |
| ESA_RS00030 | N/A | -1.16 | small subunit ribosomal protein S19 |
| ESA_RS04895 | N/A | -1.17 | elongation factor P |
| ESA_RS10990 | N/A | -1.17 | DNA transformation protein and related proteins |
| ESA_RS11105 | *aspC* | -1.17 | aspartate/tyrosine/aromatic aminotransferase |
| ESA_RS07970 | *mlc* | -1.17 | transcriptional regulator of PTS gene |
| ESA_RS08750 | N/A | -1.17 | Unknown |
| ESA_RS06045 | N/A | -1.18 | ferritin |
| ESA_RS13720 | *lacI* | -1.18 | LacI family transcriptional regulator |
| ESA_RS14585 | N/A | -1.18 | uridylate kinase |
| ESA_RS04425 | N/A | -1.19 | ribonucleoside-diphosphate reductase alpha chain |
| ESA_RS00690 | *groES* | -1.19 | chaperonin |
| ESA_RS10310 | N/A | -1.19 | 3-oxoacyl-[acyl-carrier-protein] synthase II |
| ESA_RS16475 | N/A | -1.20 | monothiol glutaredoxin |
| ESA_RS13405 | *rdgC* | -1.20 | recombination associated protein |
| ESA_RS13770 | N/A | -1.20 | Unknown |
| ESA_RS12410 | N/A | -1.20 | D-alanyl-D-alanine carboxypeptidase (penicillin-binding protein 5/6) |
| ESA_RS09900 | N/A | -1.20 | exodeoxyribonuclease III |
| ESA_RS08595 | *ansP* | -1.20 | L-asparagine permease |
| ESA_RS00685 | *fxsA* | -1.21 | UPF0716 protein |
| ESA_RS17645 | *hslV* | -1.21 | ATP-dependent HslUV protease, peptidase subunit |
| ESA_RS09220 | N/A | -1.21 | ribonuclease T |
| ESA_RS21055 | N/A | -1.21 | Unknown |
| ESA_RS07730 | N/A | -1.22 | Unknown |
| ESA_RS18395 | N/A | -1.22 | enterochelin esterase and related enzymes |
| ESA_RS04105 | N/A | -1.22 | acetyl-CoA carboxylase carboxyl transferase subunit beta |
| ESA_RS17250 | *corA* | -1.22 | magnesium/nickel/cobalt transporter |
| ESA_RS14340 | *gmhA* | -1.23 | D-sedoheptulose 7-phosphate isomerase |
| ESA_RS17170 | *rmuC* | -1.23 | DNA recombination protein |
| ESA_RS00425 | N/A | -1.23 | tRNA-dihydrouridine synthase A |
| ESA_RS11520 | *rimO* | -1.24 | ribosomal protein S12 methylthiotransferase |
| ESA_RS21130 | N/A | -1.24 | outer membrane lipase/esterase |
| ESA_RS01140 | N/A | -1.24 | L-seryl-tRNA(Ser) seleniumtransferase |
| ESA_RS12050 | *sucC* | -1.25 | succinyl-CoA synthetase beta subunit |
| ESA_RS20280 | *fkpA* | -1.25 | FKBP-type peptidyl-prolyl cis-trans isomerase |
| ESA_RS18885 | *rpmB* | -1.26 | large subunit ribosomal protein L28 |
| ESA_RS16775 | N/A | -1.26 | acetyl-CoA carboxylase, biotin carboxylase subunit |
| ESA_RS03240 | N/A | -1.27 | myo-inositol-1(or 4)-monophosphatase |
| ESA_RS13140 | N/A | -1.27 | 7-cyano-7-deazaguanine synthase |
| ESA_RS06060 | N/A | -1.28 | ribose 5-phosphate isomerase B |
| ESA_RS04880 | *cobW* | -1.28 | cobalamin biosynthesis protein |
| ESA_RS01200 | N/A | -1.29 | TetR/AcrR family transcriptional regulator, fatty acid metabolism regulator protein |
| ESA_RS00280 | *rtcR* | -1.29 | transcriptional regulatory protein |
| ESA_RS11295 | N/A | -1.30 | translation initiation factor IF-1 |
| ESA_RS11730 | N/A | -1.30 | Unknown |
| ESA_RS19315 | N/A | -1.30 | putative acetyltransferase |
| ESA_RS12250 | *nagB* | -1.30 | glucosamine-6-phosphate deaminase |
| ESA_RS12870 | *htpG* | -1.30 | molecular chaperone |
| ESA_RS02045 | N/A | -1.31 | 2-deoxy-D-gluconate 3-dehydrogenase |
| ESA_RS16740 | N/A | -1.31 | rod shape-determining protein MreB and related proteins |
| ESA_RS13505 | N/A | -1.32 | D-alanyl-D-alanine-carboxypeptidase / D-alanyl-D-alanine-endopeptidase |
| ESA_RS18825 | N/A | -1.32 | Unknown |
| ESA_RS21200 | N/A | -1.32 | chromosome partitioning protein, ParB family |
| ESA_RS12720 | N/A | -1.32 | LacI family transcriptional regulator, maltose regulon regulatory protein |
| ESA_RS16895 | N/A | -1.33 | Unknown |
| ESA_RS03875 | *glk* | -1.33 | glucokinase |
| ESA_RS04940 | N/A | -1.33 | predicted membrane protein |
| ESA_RS13495 | N/A | -1.34 | peptide/bleomycin uptake transporter |
| ESA_RS18270 | N/A | -1.34 | galactonate dehydratase |
| ESA_RS02640 | N/A | -1.34 | MarR family transcriptional regulator, negative regulator of the multidrug operon emrRAB |
| ESA_RS17655 | N/A | -1.35 | 1,4-dihydroxy-2-naphthoate octaprenyltransferase [EC:2.5.1.74 2.5.1.-] |
| ESA_RS21205 | N/A | -1.35 | chromosome partitioning protein |
| ESA_RS14990 | N/A | -1.36 | MraZ protein |
| ESA_RS17285 | *cyaY* | -1.36 | CyaY protein |
| ESA_RS07660 | N/A | -1.37 | diguanylate cyclase |
| ESA_RS07300 | N/A | -1.39 | enoyl-[acyl-carrier protein] reductase I |
| ESA_RS00750 | N/A | -1.40 | fumarate reductase flavoprotein subunit |
| ESA_RS11610 | *opgE* | -1.40 | heptose-I-phosphate ethanolaminephosphotransferase |
| ESA_RS03860 | N/A | -1.41 | Unknown |
| ESA_RS19245 | N/A | -1.42 | glycyl-tRNA synthetase alpha chain |
| ESA_RS02635 | N/A | -1.43 | membrane fusion protein, multidrug efflux system |
| ESA_RS02885 | *grpE* | -1.43 | molecular chaperone |
| ESA_RS17535 | N/A | -1.43 | NAD(P) transhydrogenase |
| ESA_RS13185 | N/A | -1.43 | trigger factor |
| ESA_RS15295 | *dnaK* | -1.44 | molecular chaperone |
| ESA_RS04195 | N/A | -1.44 | Unknown |
| ESA_RS12855 | N/A | -1.44 | gluconate:H+ symporter, GntP family |
| ESA_RS19320 | N/A | -1.44 | DNA-3-methyladenine glycosylase I |
| ESA_RS15695 | N/A | -1.44 | membrane fusion protein, multidrug efflux system |
| ESA_RS03520 | N/A | -1.44 | arsenate reductase |
| ESA_RS12860 | N/A | -1.45 | carbohydrate diacid regulator |
| ESA_RS12360 | N/A | -1.45 | methyl-accepting chemotaxis protein II, aspartate sensor receptor |
| ESA_RS10565 | N/A | -1.45 | Unknown |
| ESA_RS19610 | N/A | -1.46 | murein DD-endopeptidase |
| ESA_RS18275 | N/A | -1.48 | 2-dehydro-3-deoxyphosphogalactonate aldolase |
| ESA_RS12595 | *trg* | -1.48 | methyl-accepting chemotaxis protein |
| ESA_RS08480 | *nlpC* | -1.49 | probable lipoprotein |
| ESA_RS13025 | N/A | -1.49 | Unknown |
| ESA_RS06125 | N/A | -1.50 | universal stress protein C |
| ESA_RS03045 | N/A | -1.50 | acetyltransferase |
| ESA_RS14580 | *frr* | -1.50 | ribosome recycling factor |
| ESA_RS01835 | N/A | -1.50 | putative metalloprotease |
| ESA_RS00675 | N/A | -1.51 | anaerobic C4-dicarboxylate transporter DcuA |
| ESA_RS19440 | N/A | -1.51 | zinc protease |
| ESA_RS18690 | N/A | -1.51 | sulfoquinovose isomerase |
| ESA_RS15485 | *deoD* | -1.51 | purine-nucleoside phosphorylase |
| ESA_RS18625 | N/A | -1.52 | GTP-binding protein |
| ESA_RS17635 | N/A | -1.53 | LacI family transcriptional regulator, repressor for deo operon, udp, cdd, tsx, nupC, and nupG |
| ESA_RS13370 | *proY* | -1.53 | proline-specific permease |
| ESA_RS02645 | N/A | -1.54 | Unknown |
| ESA_RS00710 | N/A | -1.54 | elongation factor P |
| ESA_RS18860 | *pyrE* | -1.55 | orotate phosphoribosyltransferase |
| ESA_RS07360 | N/A | -1.55 | multiple sugar transport system substrate-binding protein |
| ESA_RS04395 | N/A | -1.56 | Unknown |
| ESA_RS03870 | N/A | -1.56 | indolepyruvate decarboxylase |
| ESA_RS00890 | *aidB* | -1.56 | isovaleryl-CoA dehydrogenase |
| ESA_RS15580 | *chbC* | -1.56 | PTS cellobiose transporter subunit IIC |
| ESA_RS04875 | *lpxT* | -1.57 | lipid A 1-diphosphate synthase |
| ESA_RS18325 | N/A | -1.57 | YidC/Oxa1 family membrane protein insertase |
| ESA_RS08870 | N/A | -1.57 | small multidrug resistance pump |
| ESA_RS04005 | N/A | -1.58 | long-chain fatty acid transport protein |
| ESA_RS12060 | N/A | -1.58 | 2-oxoglutarate dehydrogenase E1 component |
| ESA_RS18320 | *rnpA* | -1.59 | ribonuclease P protein component |
| ESA_RS04265 | N/A | -1.61 | NADH-quinone oxidoreductase subunit C/D |
| ESA_RS15400 | *ettA* | -1.61 | energy-dependent translational throttle protein |
| ESA_RS04070 | N/A | -1.62 | 3-oxoacyl-[acyl-carrier-protein] synthase I |
| ESA_RS17680 | N/A | -1.63 | ribosome-associated protein |
| ESA_RS00550 | *acs* | -1.65 | acetyl-coenzyme A synthetase |
| ESA_RS12450 | N/A | -1.66 | cold shock protein (beta-ribbon, CspA family) |
| ESA_RS16235 | N/A | -1.67 | glycosyl hydrolase family 32 |
| ESA_RS15725 | N/A | -1.70 | arabinogalactan endo-1,4-beta-galactosidase |
| ESA_RS09645 | *ppsA* | -1.70 | phosphoenolpyruvate synthase |
| ESA_RS18710 | N/A | -1.71 | DeoR family transcriptional regulator, glycerol-3-phosphate regulon repressor |
| ESA_RS19340 | *eptB* | -1.72 | kdo(2)-lipid A phosphoethanolamine 7''-transferase |
| ESA_RS13215 | N/A | -1.72 | cytochrome o ubiquinol oxidase subunit II |
| ESA_RS17820 | N/A | -1.72 | FdhE protein |
| ESA_RS14705 | *GluQ* | -1.73 | tRNA glutamyl-Q(34) synthetase |
| ESA_RS09190 | N/A | -1.73 | aryl-alcohol dehydrogenase (NADP+) |
| ESA_RS05170 | N/A | -1.73 | Unknown |
| ESA_RS08760 | *trg* | -1.73 | methyl-accepting chemotaxis protein |
| ESA_RS07095 | N/A | -1.73 | Unknown |
| ESA_RS07880 | N/A | -1.74 | para-nitrobenzyl esterase |
| ESA_RS21050 | N/A | -1.74 | Unknown |
| ESA_RS06835 | N/A | -1.75 | ribose-phosphate pyrophosphokinase |
| ESA_RS14995 | *cra* | -1.75 | catabolite repressor/activator |
| ESA_RS01935 | *gcvP* | -1.76 | glycine dehydrogenase |
| ESA_RS15180 | N/A | -1.76 | RpiR family transcriptional regulator, carbohydrate utilization regulator |
| ESA_RS18545 | N/A | -1.77 | ribose transport system substrate-binding protein |
| ESA_RS01775 | N/A | -1.77 | Unknown |
| ESA_RS12715 | *ptsG* | -1.79 | PTS system, glucose-specific IIB component |
| ESA_RS07820 | N/A | -1.80 | lactaldehyde dehydrogenase / glycolaldehyde dehydrogenase |
| ESA_RS13030 | N/A | -1.80 | Unknown |
| ESA_RS04390 | *tctE* | -1.81 | two-component system, OmpR family, sensor histidine kinase |
| ESA_RS06550 | *manZ* | -1.82 | PTS mannose transporter subunit IID |
| ESA_RS01475 | *trmB* | -1.83 | tRNA (guanine-N7-)-methyltransferase |
| ESA_RS16970 | N/A | -1.83 | AraC family transcriptional regulator, regulatory protein of adaptative response / DNA-3-methyladenine glycosylase II |
| ESA_RS04020 | *fadJ* | -1.83 | 3-hydroxyacyl-CoA dehydrogenase / enoyl-CoA hydratase / 3-hydroxybutyryl-CoA epimerase |
| ESA_RS03160 | *murP* | -1.84 | PTS sugar transporter subunit IIC |
| ESA_RS18200 | *emrD* | -1.84 | multidrug transporter |
| ESA_RS06090 | N/A | -1.84 | ferritin-like protein 2 |
| ESA_RS09375 | *betT* | -1.85 | high-affinity choline transporter |
| ESA_RS04260 | N/A | -1.86 | NADH-quinone oxidoreductase subunit B |
| ESA_RS19850 | N/A | -1.86 | scyllo-inositol 2-dehydrogenase (NADP+) |
| ESA_RS15205 | N/A | -1.88 | alanyl-tRNA synthetase |
| ESA_RS12065 | *sdhB* | -1.90 | succinate dehydrogenase / fumarate reductase, iron-sulfur subunit |
| ESA_RS19210 | *xylF* | -1.91 | D-xylose transport system substrate-binding protein |
| ESA_RS00390 | N/A | -1.91 | diacylglycerol kinase (ATP) |
| ESA_RS17625 | *rpmE* | -1.91 | large subunit ribosomal protein L31 |
| ESA_RS15135 | *folA* | -1.92 | dihydrofolate reductase |
| ESA_RS01635 | N/A | -1.92 | SH3 domain protein |
| ESA_RS08745 | N/A | -1.93 | Unknown |
| ESA_RS16770 | N/A | -1.93 | acetyl-CoA carboxylase biotin carboxyl carrier protein |
| ESA_RS03155 | *murQ* | -1.95 | N-acetylmuramic acid 6-phosphate etherase |
| ESA_RS02125 | *galR* | -1.95 | HTH-type transcriptional regulator |
| ESA_RS04385 | *tctD* | -1.95 | transcriptional regulator |
| ESA_RS13075 | N/A | -1.95 | methylated-DNA--protein-cysteine methyltransferase |
| ESA_RS09390 | *betA* | -1.95 | oxygen-dependent choline dehydrogenase |
| ESA_RS00365 | *lamB* | -1.97 | maltoporin |
| ESA_RS12520 | *uspG* | -2.01 | universal stress protein G |
| ESA_RS12230 | *chiP* | -2.01 | chitoporin |
| ESA_RS20840 | N/A | -2.01 | Unknown |
| ESA_RS08740 | *srfB* | -2.01 | virulence factor SrfB |
| ESA_RS01505 | *yjiA* | -2.02 | GTPase |
| ESA_RS11705 | *bglG* | -2.02 | hypothetical protein |
| ESA_RS16050 | *aer* | -2.04 | aerotaxis receptor |
| ESA_RS01830 | *speB* | -2.05 | agmatinase |
| ESA_RS06555 | *manY* | -2.05 | PTS mannose/fructose/sorbose transporter subunit IIC |
| ESA_RS15245 | *rpsT* | -2.06 | 30S ribosomal protein S20 |
| ESA_RS12470 | *menH* | -2.07 | hypothetical protein |
| ESA_RS13645 | *gcd* | -2.10 | membrane-bound PQQ-dependent dehydrogenase |
| ESA_RS20130 | *frlR* | -2.10 | GntR family transcriptional regulator |
| ESA_RS18705 | *yihV* | -2.11 | ribokinase |
| ESA_RS16595 | *yhcH* | -2.11 | YhcH/YjgK/YiaL family protein |
| ESA_RS01125 | N/A | -2.12 | membrane protein |
| ESA_RS02290 | *gcvH* | -2.13 | glycine cleavage system protein H |
| ESA_RS01605 | *rpsU* | -2.14 | 30S ribosomal protein S21 |
| ESA_RS16110 | *uxaC* | -2.14 | uronate isomerase |
| ESA_RS08920 | *ybiV* | -2.14 | sugar-phosphatase |
| ESA_RS15705 | *lacI* | -2.16 | lac repressor |
| ESA_RS01590 | *csgG* | -2.18 | hypothetical protein |
| ESA_RS00350 | *malF* | -2.18 | maltose ABC transporter permease MalF |
| ESA_RS08060 | *ydfI* | -2.18 | D-mannonate oxidoreductase |
| ESA_RS05535 | *dacD* | -2.18 | D-alanyl-D-alanine carboxypeptidase |
| ESA_RS07840 | N/A | -2.18 | hypothetical protein |
| ESA_RS02370 | *nanE* | -2.19 | N-acetylmannosamine-6-phosphate 2-epimerase |
| ESA_RS02100 | *chbC* | -2.20 | PTS lactose transporter subunit IIC |
| ESA_RS15730 | *malG* | -2.23 | arabinogalactan ABC transporter permease |
| ESA_RS16105 | *uxaA* | -2.23 | altronate hydrolase |
| ESA_RS13375 | *brnQ* | -2.24 | branched-chain amino acid transporter 2 carrier protein BrnQ |
| ESA_RS02040 | *tolB* | -2.25 | oligogalacturonate lyase |
| ESA_RS15065 | *araC* | -2.26 | DNA-binding transcriptional regulator AraC |
| ESA_RS18265 | *dgoT* | -2.28 | MFS transporter |
| ESA_RS20745 | N/A | -2.28 | Unknown |
| ESA_RS18695 | *yihT* | -2.32 | aldolase |
| ESA_RS15715 | *ptsG* | -2.32 | PTS sugar transporter |
| ESA_RS01130 | N/A | -2.32 | membrane protein |
| ESA_RS01135 | N/A | -2.33 | dihydroorotase |
| ESA_RS15720 | N/A | -2.33 | beta-galactosidase |
| ESA_RS14755 | *hpt* | -2.33 | hypoxanthine phosphoribosyltransferase |
| ESA_RS03340 | *ndk* | -2.34 | nucleoside-diphosphate kinase |
| ESA_RS19195 | *xylR* | -2.34 | XylR family transcriptional regulator |
| ESA_RS01885 | *rpiA* | -2.34 | ribose-5-phosphate isomerase |
| ESA_RS14345 | *fadE* | -2.35 | acyl-CoA dehydrogenase |
| ESA_RS09385 | *betB* | -2.35 | betaine-aldehyde dehydrogenase |
| ESA_RS06560 | *manX* | -2.36 | PTS mannose transporter subunit EIIAB |
| ESA_RS04170 | *yfcH* | -2.37 | epimerase |
| ESA_RS12080 | *sdhC* | -2.38 | succinate dehydrogenase cytochrome b556 large subunit |
| ESA_RS03795 | N/A | -2.39 | FlxA protein |
| ESA_RS01805 | *endA* | -2.39 | deoxyribonuclease I |
| ESA_RS05155 | *yehT* | -2.39 | DNA-binding response regulator |
| ESA_RS00355 | *malE* | -2.40 | maltose ABC transporter substrate-binding protein MalE |
| ESA_RS17800 | N/A | -2.40 | sulfate ABC transporter substrate-binding protein |
| ESA_RS04890 | *uxuA* | -2.41 | mannonate dehydratase |
| ESA_RS07855 | *trg* | -2.41 | methyl-accepting chemotaxis protein |
| ESA_RS16780 | *yhdT* | -2.42 | membrane protein |
| ESA_RS05165 | N/A | -2.43 | hypothetical protein |
| ESA_RS12070 | *sdhA* | -2.44 | succinate dehydrogenase flavoprotein subunit |
| ESA_RS18880 | *yicR* | -2.44 | hypothetical protein |
| ESA_RS05960 | N/A | -2.46 | acyl-CoA reductase |
| ESA_RS14315 | *gpt* | -2.46 | xanthine phosphoribosyltransferase |
| ESA_RS08960 | *ydgI* | -2.46 | arginine:ornithine antiporter |
| ESA_RS18230 | *yidP* | -2.48 | GntR family transcriptional regulator |
| ESA_RS01110 | *glrR* | -2.49 | glycine dehydrogenase |
| ESA_RS19950 | *malT* | -2.50 | transcriptional regulator |
| ESA_RS12075 | *sdhD* | -2.50 | succinate dehydrogenase cytochrome b556 small membrane subunit |
| ESA_RS19755 | *puuE* | -2.52 | aspartate aminotransferase family protein |
| ESA_RS09270 | *ascB* | -2.53 | 6-phospho-beta-glucosidase |
| ESA_RS03630 | *cstA* | -2.57 | carbon starvation protein A |
| ESA_RS16230 | *treB* | -2.58 | PTS sucrose IIB component / PTS sucrose IIC component |
| ESA_RS18700 | *yihU* | -2.59 | NADH-dependent gamma-hydroxybutyrate dehydrogenase |
| ESA_RS09265 | *chbC* | -2.59 | PTS cellobiose transporter subunit IIC |
| ESA_RS03575 | *maeB* | -2.60 | bifunctional malic enzyme oxidoreductase/phosphotransacetylase |
| ESA_RS02365 | N/A | -2.62 | PTS glucose transporter subunit IIB |
| ESA_RS19280 | *lgoT* | -2.62 | MFS transporter |
| ESA_RS05930 | N/A | -2.63 | hypothetical protein |
| ESA_RS02075 | N/A | -2.64 | cupin |
| ESA_RS01075 | *tar* | -2.65 | methyl-accepting chemotaxis protein |
| ESA_RS19495 | N/A | -2.65 | hypothetical protein |
| ESA_RS04885 | *yeiQ* | -2.69 | D-mannonate oxidoreductase |
| ESA_RS19290 | *yihM* | -2.69 | hypothetical protein |
| ESA_RS06545 | *yobD* | -2.69 | hypothetical protein |
| ESA_RS16065 | *fadH* | -2.69 | NADPH-dependent 2,4-dienoyl-CoA reductase |
| ESA_RS09380 | *betI* | -2.70 | transcriptional regulator |
| ESA_RS01415 | *citA* | -2.70 | sensor histidine kinase |
| ESA_RS05000 | *cdd* | -2.70 | cytidine deaminase |
| ESA_RS17045 | *coaA* | -2.71 | type I pantothenate kinase |
| ESA_RS09060 | *malY* | -2.71 | pyridoxal phosphate-dependent aminotransferase |
| ESA_RS01120 | N/A | -2.71 | hypothetical protein |
| ESA_RS04415 | *yfaE* | -2.71 | (2Fe-2S) ferredoxin |
| ESA_RS08735 | N/A | -2.72 | hypothetical protein |
| ESA_RS00370 | *malM* | -2.74 | maltose operon protein MalM |
| ESA_RS06375 | *hexR* | -2.75 | transcriptional regulator HexR |
| ESA_RS06185 | *tar* | -2.75 | methyl-accepting chemotaxis protein |
| ESA_RS01300 | *chbC* | -2.77 | PTS cellobiose transporter subunit IIC |
| ESA_RS02030 | *yqeF* | -2.77 | acetyl-CoA acetyltransferase |
| ESA_RS00720 | N/A | -2.78 | helix-turn-helix transcriptional regulator |
| ESA_RS17760 | *lacY* | -2.78 | MFS transporter |
| ESA_RS09000 | *fumA* | -2.82 | fumarate hydratase |
| ESA_RS01055 | *ytfQ* | -2.82 | sugar ABC transporter substrate-binding protein |
| ESA_RS15495 | *deoA* | -2.84 | thymidine phosphorylase |
| ESA_RS05920 | *arnB* | -2.85 | DegT/DnrJ/EryC1/StrS family aminotransferase |
| ESA_RS05150 | *yehU* | -2.87 | Autolysin sensor kinase |
| ESA_RS16965 | *chbA* | -2.89 | PTS lactose/cellobiose transporter subunit IIA |
| ESA_RS06720 | *dadA* | -2.90 | D-amino acid dehydrogenase small subunit |
| ESA_RS05955 | N/A | -2.94 | acyl-protein synthetase |
| ESA_RS01410 | *dpiA* | -2.94 | two-component system response regulator |
| ESA_RS15500 | *deoC* | -2.95 | 2-deoxyribose-5-phosphate aldolase |
| ESA_RS01290 | *lamB* | -2.95 | carbohydrate porin |
| ESA_RS17105 | *fadA* | -2.96 | acetyl-CoA C-acyltransferase FadA |
| ESA_RS09880 | *astB* | -2.97 | succinylarginine dihydrolase |
| ESA_RS03865 | *gpr* | -2.98 | glyceraldehyde 3-phosphate reductase |
| ESA_RS06725 | *dadX* | -2.99 | alanine racemase |
| ESA_RS05950 | *fadK* | -3.01 | hypothetical protein |
| ESA_RS09055 | *malX* | -3.01 | PTS maltose transporter subunit IICB |
| ESA_RS12510 | *rnk* | -3.04 | nucleoside diphosphate kinase regulator |
| ESA_RS19930 | *glpD* | -3.04 | glycerol-3-phosphate dehydrogenase |
| ESA_RS01925 | *gcvT* | -3.06 | aminomethyltransferase |
| ESA_RS05915 | *rsmC* | -3.08 | hypothetical protein |
| ESA_RS05940 | *fabG* | -3.09 | 3-oxoacyl-ACP reductase |
| ESA_RS15505 | *nupX* | -3.11 | NupC/NupG family nucleoside CNT transporter |
| ESA_RS16225 | *lamB* | -3.12 | sucrose porin |
| ESA_RS05935 | *fabG* | -3.12 | NAD(P)-dependent oxidoreductase |
| ESA_RS07355 | *ycjM* | -3.13 | sugar phosphorylase |
| ESA_RS05925 | *lpxA* | -3.13 | sugar O-acyltransferase |
| ESA_RS16605 | *nanT* | -3.17 | MFS transporter |
| ESA_RS19705 | *zntA* | -3.22 | zinc/cadmium/mercury/lead-transporting ATPase |
| ESA_RS05910 | N/A | -3.25 | hypothetical protein |
| ESA_RS15735 | N/A | -3.25 | sugar ABC transporter permease |
| ESA_RS06820 | *ychH* | -3.26 | membrane protein |
| ESA_RS01005 | *ytfJ* | -3.26 | YtfJ family protein |
| ESA_RS19285 | *ydjH* | -3.35 | sugar kinase |
| ESA_RS15745 | N/A | -3.36 | ABC transporter ATP-binding protein |
| ESA_RS09875 | *astE* | -3.38 | succinylglutamate desuccinylase |
| ESA_RS03850 | *nupC* | -3.40 | NupC/NupG family nucleoside CNT transporter |
| ESA_RS01510 | *yjiX* | -3.42 | DUF466 domain-containing protein |
| ESA_RS09125 | *dtpA* | -3.45 | dipeptide/tripeptide permease A |
| ESA_RS07875 | *ydcJ* | -3.45 | DUF1338 domain-containing protein |
| ESA_RS02105 | *bglA* | -3.48 | 6-phospho-beta-glucosidase |
| ESA_RS17100 | *fadB* | -3.51 | fatty acid oxidation complex subunit alpha FadB |
| ESA_RS15710 | *lamB* | -3.55 | maltoporin |
| ESA_RS05795 | *umuD* | -3.60 | hypothetical protein |
| ESA_RS13715 | *lacZ* | -3.61 | beta-galactosidase |
| ESA_RS07705 | *ygeV* | -3.62 | sigma-54-dependent Fis family transcriptional regulator |
| ESA_RS06050 | *yecR* | -3.63 | hypothetical protein |
| ESA_RS10835 | *putA* | -3.65 | trifunctional transcriptional regulator/proline dehydrogenase/L-glutamate gamma-semialdehyde dehydrogenase |
| ESA_RS01115 | N/A | -3.66 | hypothetical protein |
| ESA_RS16600 | *nanK* | -3.68 | N-acetylmannosamine kinase |
| ESA_RS02110 | *chbB* | -3.90 | PTS sugar transporter subunit IIB |
| ESA_RS19435 | *dctA* | -3.95 | C4-dicarboxylate transporter |
| ESA_RS00995 | *cpdB* | -3.95 | 2',3'-cyclic-nucleotide 2'-phosphodiesterase |
| ESA_RS09885 | *astD* | -3.95 | N-succinylglutamate 5-semialdehyde dehydrogenase |
| ESA_RS21355 | *astA* | -3.96 | arginine N-succinyltransferase |
| ESA_RS06575 | *sdaA* | -3.96 | L-serine ammonia-lyase |
| ESA_RS18970 | *tdh* | -4.07 | L-threonine 3-dehydrogenase |
| ESA_RS17705 | *lidD* | -4.12 | alpha-hydroxy-acid oxidizing enzyme |
| ESA_RS01180 | *kefG* | -4.13 | oxidoreductase |
| ESA_RS20025 | *pck* | -4.20 | phosphoenolpyruvate carboxykinase (ATP) |
| ESA_RS16610 | *nanA* | -4.30 | N-acetylneuraminate lyase |
| ESA_RS09760 | *eco* | -4.31 | ecotin |
| ESA_RS17765 | N/A | -4.32 | alpha-galactosidase |
| ESA_RS04975 | *galS* | -4.33 | DNA-binding transcriptional regulator GalS |
| ESA_RS15740 | N/A | -4.35 | cyclodextrin-binding protein |
| ESA_RS17815 | N/A | -4.41 | formate dehydrogenase cytochrome b556 subunit |
| ESA_RS17725 | *rhaR* | -4.43 | HTH-type transcriptional activator RhaR |
| ESA_RS17805 | *fdoG* | -4.48 | formate dehydrogenase-N subunit alpha |
| ESA_RS04455 | *phoE* | -4.49 | phosphoporin PhoE |
| ESA_RS17810 | *fdoH* | -4.62 | formate dehydrogenase subunit beta |
| ESA_RS15645 | *tsr* | -4.65 | methyl-accepting chemotaxis protein |
| ESA_RS00360 | *malK* | -4.79 | ABC transporter ATP-binding protein |
| ESA_RS17175 | *udp* | -4.81 | uridine phosphorylase |
| ESA_RS18965 | *kbl* | -4.83 | glycine C-acetyltransferase |
| ESA_RS02290 | *sdaC* | -4.69 | serine/threonine transporter |
